# Correlation of triglyceride-glucose index with the incidence and prognosis of hyperglycemic crises in critically ill patients with diabetes mellitus: a machine-learning-based multicenter retrospective cohort study

**DOI:** 10.3389/fnut.2025.1649553

**Published:** 2025-09-04

**Authors:** Mingchen Xie, Yahui Zhang, Haitao Wu, Zeyu Wu, Hao Han, Xun Xie, Rui Zhang, Jianhua Cheng, Jian Xu

**Affiliations:** ^1^Department of Neurosurgery (Diabetes Critical Care Research Consortium), The Affiliated Hospital of Qingdao University, Qingdao, China; ^2^Department of Neurological Intensive Care Unit, The Affiliated Hospital of Qingdao University, Qingdao, China; ^3^Qingdao University Medical College, Qingdao University, Qingdao, China

**Keywords:** triglyceride-glucose index, hyperglycemic crisis, critical care, machine learning, mortality prediction

## Abstract

**Background:**

Hyperglycemic crisis events (HCEs)—encompassing diabetic ketoacidosis (DKA) and hyperosmolar hyperglycemic state (HHS)—constitute lethal determinants for patients with diabetic mellitus (DM) in intensive care. The triglyceride-glucose (TyG) index, an emergent insulin resistance surrogate, lacks rigorous investigation regarding HCE occurrence trajectories and prognostic sequelae among critically ill diabetics. This study aims to evaluate the relationship between the TyG index and HCE incidence/clinical outcomes in critically ill patients with DM and to construct a risk prediction model using machine-learning algorithms.

**Methods:**

This multi-center retrospective investigation leveraged clinical repositories from Medical Information Mart for Intensive Care IV (MIMIC-IV) and eICU Collaborative Research Database (eICU-CRD). Inclusion criteria encompassed critically ill subjects with diabetes possessing computable TyG indices within 24 h post-admission. The main study endpoints included death occurring during hospitalization and death within the intensive care unit. TyG index-outcome interrelationships underwent interrogation via logistic regression, restricted cubic spline (RCS), correlation, and linear analytical methodologies. Overlap weighting (OW), inverse probability treatment weighting (IPTW), and propensity score matching (PSM) mitigated confounding influences. Stratified examinations occurred per determinant factors. Five machine-learning architectures constructed mortality prognostication frameworks, with SHapley Additive exPlanations (SHAP) delineating pivotal predictors.

**Results:**

Among 4,098 critically ill patients with DM, 328 developed HCE. Patients with HCE had significantly higher TyG levels [10.2 (9.6–11.0) vs. 9.4 (8.9–9.9)] than non-HCE patients, demonstrating TyG’s discriminative ability for HCE. Through multivariate logistic regression, TyG was pinpointed as a separate risk element for both in-hospital (OR 1.956) and ICU death (OR 2.260), linked to extended hospital stays. RCS established a direct positive correlation between increased TyG levels and death rates (nonlinear *p* = 0.161 and 0.457), continuing even after adjusting for PSM, OW, and IPTW. Subgroup analyses reinforced TyG’s consistent mortality correlation. Machine-learning models, particularly XGBoost, achieved higher predictive accuracy, with TyG as a key component.

**Conclusion:**

Elevated TyG index shows a notable correlation with the occurrence of HCE and negative results in critically ill patients with DM. Advanced multivariate machine-learning models are adept at pinpointing patients at high risk, thereby facilitating prompt clinical action.

## Graphical abstract

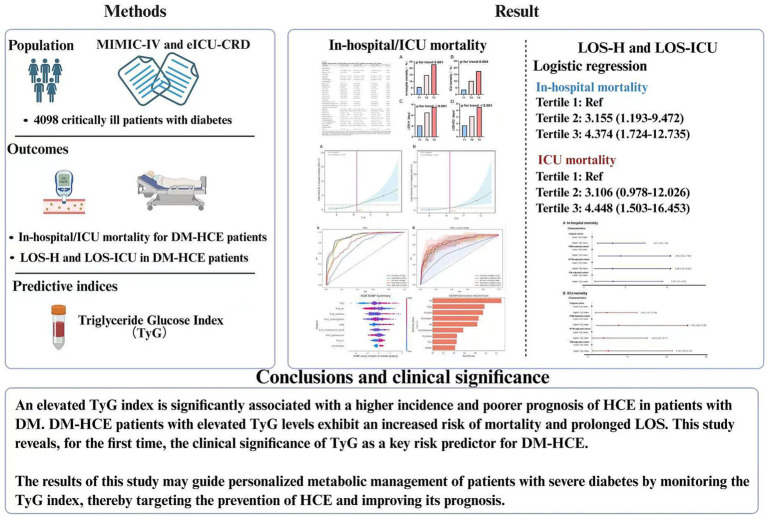

## Introduction

The hyperglycemic crisis event (HCE), encompassing conditions like diabetic ketoacidosis (DKA) and hyperosmolar hyperglycemic state (HHS), stands as the gravest immediate metabolic issue in patients with diabetes mellitus (DM) (1), with a mortality rate within hospitals reaching 5–20% (2–6). These crises frequently stem from a mix of various elements, such as infections, disruptions in insulin treatment, and metabolic strain (4, 7). Particularly in severely ill patients with DM, the likelihood of HCE escalates markedly because of the metabolic imbalances of the condition and the immediate stress condition. However, there is a lack of effective early warning indicators in clinical practice, and traditional prediction methods mostly rely on laboratory test results after the onset of the crisis, making it difficult to provide a sufficient time window for preventive intervention (8–11). Consequently, investigating dependable biomarkers for early detection of high-risk patients holds clinical importance.

The triglyceride-glucose (TyG) index has garnered extensive recognition recently as an emergent insulin resistance metric (12–15). Synthesizing fasting triglycerides and blood glucose concentrations, this indicator offers enhanced holistic characterization of organismal metabolic dysregulation (16, 17). Current evidence substantiates TyG’s robust correlation not merely with type 2 diabetes onset but also its predictive efficacy regarding chronic sequelae including cardiovascular pathologies (18, 19). Nevertheless, its prognostic utility concerning acute metabolic decompensation, specifically HCE, remains inadequately investigated. Concurrently, critical care informatics evolution and large-scale public repository establishment—notably MIMIC-IV and eICU-CRD—have furnished indispensable investigative substrates (12, 20, 21). These repositories capture expansive clinical parameters, laboratory surveillance metrics, plus granular therapeutic and prognostic documentation, enabling a profound exploration of metabolic indicator-outcome relationships.

The study utilized a retrospective cohort method across multiple centers. Preliminary studies employed logistic regression to evaluate how the TyG index independently correlates with both the occurrence and clinical results of HCE. Subsequently, we utilized machine-learning techniques to develop a predictive model that amalgamated clinical indices, lab parameters, and details of complications. Compared with traditional statistical methods, machine learning can better handle nonlinear relationships and interactions in high-dimensional data, which is expected to improve the accuracy of prediction (22, 23). The study’s findings aim to broaden the comprehension of the TyG index’s clinical significance and offer a useful instrument for risk evaluation in clinical settings, facilitating early detection and accurate treatment for HCE.

## Methods

### Study design and population

This multi-institutional retrospective observational cohort investigation sought to evaluate TyG index associations with HCE incidence and clinical trajectories among critically ill patients with diabetes, plus construct a multivariable predictor integrating indicators via machine-learning algorithms. We compiled 4,098 admission records from MIMIC-IV and eICU-CRD repositories. All diagnoses underwent dual corroboration through ICD-9/10 classification coding and clinical assessment benchmarks (Supplementary Table S1) (24). Post-screening for critical illness diabetes confirmation, exclusion criteria comprised: (1) age <18 years; (2) absent fasting glucose or triglyceride documentation within 24 h post-admission; (3) gestational or secondary DM (e.g., pancreatic origin). For recurrent admissions, solely, the initial ICU records underwent extraction.

The study analysis consisted of three main parts: first, the difference in TyG levels between critically ill patients with DM who developed HCE and those who did not was examined. Next, subgroup analyses of the DM-HCE population were performed based on TyG levels or mortality status. Ultimately, the application of machine-learning techniques led to the development of predictive models that amalgamate various indicators.

### Data source

MIMIC-IV constitutes a publicly available repository originating solely from Beth Israel Deaconess Medical Center, housing exhaustive high-fidelity clinical records for ICU admissions. This investigation incorporated patient histories, diagnostic evaluations, and therapeutic measures spanning 2008–2019 (20). BIDMC’s Institutional Review Board provided ethics authorization, dispensing with individual consent mandates. Researcher MCX executed dataset curation.

The eICU-CRD serves as an extensive database, encompassing clinical data from more than 200,000 admissions to intensive care units in 208 U.S. healthcare institutions. In this research, scientists employed patient information gathered from 2014 to 2015, which included comprehensive records of physiological factors, diagnostic test results, and drug therapies. This openly available database is maintained through official channels. The data collection was conducted by investigator MCX.

### Data extraction

Data acquisition deployed Navicat Premium (v16.3.11). Extracted datasets encompassed: (a) Anthropometric records: age, gender; (b) physiological metrics: pulse rate, respiration frequency, mean arterial pressure within initial hospitalization day; (c) comorbidity documentation: hypertension (HTN), hyperlipidemia (HLP), chronic obstructive pulmonary disease (COPD), heart failure (HF), ischemic heart disease (IHD), myocardial infarction (MI), acute kidney injury (AKI), chronic kidney disease (CKD), and sepsis; (d) laboratory data within 24 h of admission, including hemoglobin (Hb), platelets (Plt), red blood cells (RBC), white blood cells (WBC), potassium, sodium, creatinine, blood urea nitrogen, high-density lipoprotein cholesterol (HDL-C), total cholesterol (TC), triglycerides (TG), and fasting blood glucose; (e) treatments during hospitalization, including glucocorticoids, hypoglycemic therapy, and mechanical ventilation; (f) disease severity scores, such as the Sequential Organ Failure Assessment (SOFA) score. Personal identifiers remained undisclosed. TyG index computation employed: lnfasting TG (mg/dL) * FBG (mg/dL)/2 (25).

### Endpoints

Key outcomes focused on mortality within the hospital and in the ICU among individuals suffering from DM-HCE. The secondary goals encompassed the length of stay in hospitalization (LOS-H) and the length of stay in the ICU (LOS-ICU).

### Statistical analysis

Analytical procedures utilized R software, GraphPad Prism, and DecisionLinnc software (26). Continuous variable normality underwent Shapiro–Wilk evaluation: normally distributed parameters appeared as mean ± SD, subjected to Student’s *t*-test or one-way ANOVA; non-normal variables manifested as median (IQR), analyzed via Wilcoxon rank-sum testing. Categorical elements emerged as frequency counts (percentages), examined through *χ*^2^ testing or Fisher’s exact methodology. Multicollinearity assessment employed variance inflation factors (VIF), with VIF >5 triggering variable exclusion from multivariate modeling (27). Variables exceeding 10% missing data underwent omission. For 5–10% missing value prevalence, multiple imputation predicted absent values utilizing optimal datasets. Parameters with under 5% missingness received mean value substitution. Statistical significance threshold remained *α* = 0.05.

Mortality risk associations with the TyG index underwent scrutiny via univariate and multivariate logistic regression frameworks. Analyses treated the TyG index as a categorical or continuous metric. Confounding variables underwent selection predicated upon statistically significant baseline characteristic disparities and acknowledged clinical pertinence. Mortality and LOS trajectories underwent assessment through the TyG index ordinal categorization. Restricted cubic spline (RCS) curves probed potential nonlinear death risk relationships among patients with DM-HCE. Post-stratification of the aggregate DM-HCE cohort per RCS-derived critical thresholds (TyG index <10.2 or ≥10.2), propensity score matching (PSM) protocols, inverse probability treatment weighting (IPTW) adjustments, and overlap weighting (OW) methodologies implemented additional population variation rectification and bias mitigation. Multivariate linear regression alongside correlation assessment evaluated TyG linkages with clinical continuity markers—encompassing LOS and SOFA scores. AUC disparities underwent comparative analysis via DeLong testing. Subgroup stratification occurred by sex, age, comorbidity status, and SOFA severity. Multivariate logistic regression models deployed comprehensive Model 3 covariate adjustment excluding stratification variables. Conclusively, sensitivity analysis evaluated study robustness through exclusionary criteria: subjects manifesting incomplete covariate documentation or ≥1 hypoglycemic episode.

### Construction and performance evaluation of machine-learning models

To ascertain paramount predictive factors, Lasso regression implemented feature selection. Study subjects underwent randomized partition into dual cohorts: 80% designated training, 20% testing. Utilizing identified variables, five distinct machine-learning architectures were engineered: Decision Tree (DT), Random Forest (RF), Extreme Gradient Boosting (XGB), Support Vector Machine (SVM), and Light Gradient Boosting Machine (LGB). Fivefold cross-validation mitigated potential bias from singular data partitioning while furnishing enhanced model performance robustness.

Model efficacy underwent appraisal via multiple metrics—receiver operating characteristic curve area (AUROC), specificity, sensitivity, accuracy, and *F*_1_-score—with AUROC constituting the predominant evaluative benchmark. The premier model emerged as this investigation’s pivotal predictive instrument. Calibration scrutiny probed congruence between empirical outcomes and projected results; decision curve analysis (DCA) quantified clinical applicability. Supplementary interpretability exploration occurred through SHapley Additive exPlanations (SHAP) value analysis (28).

## Results

### Elevated TyG index observed in DM population experiencing HCE

**Figure 1:**
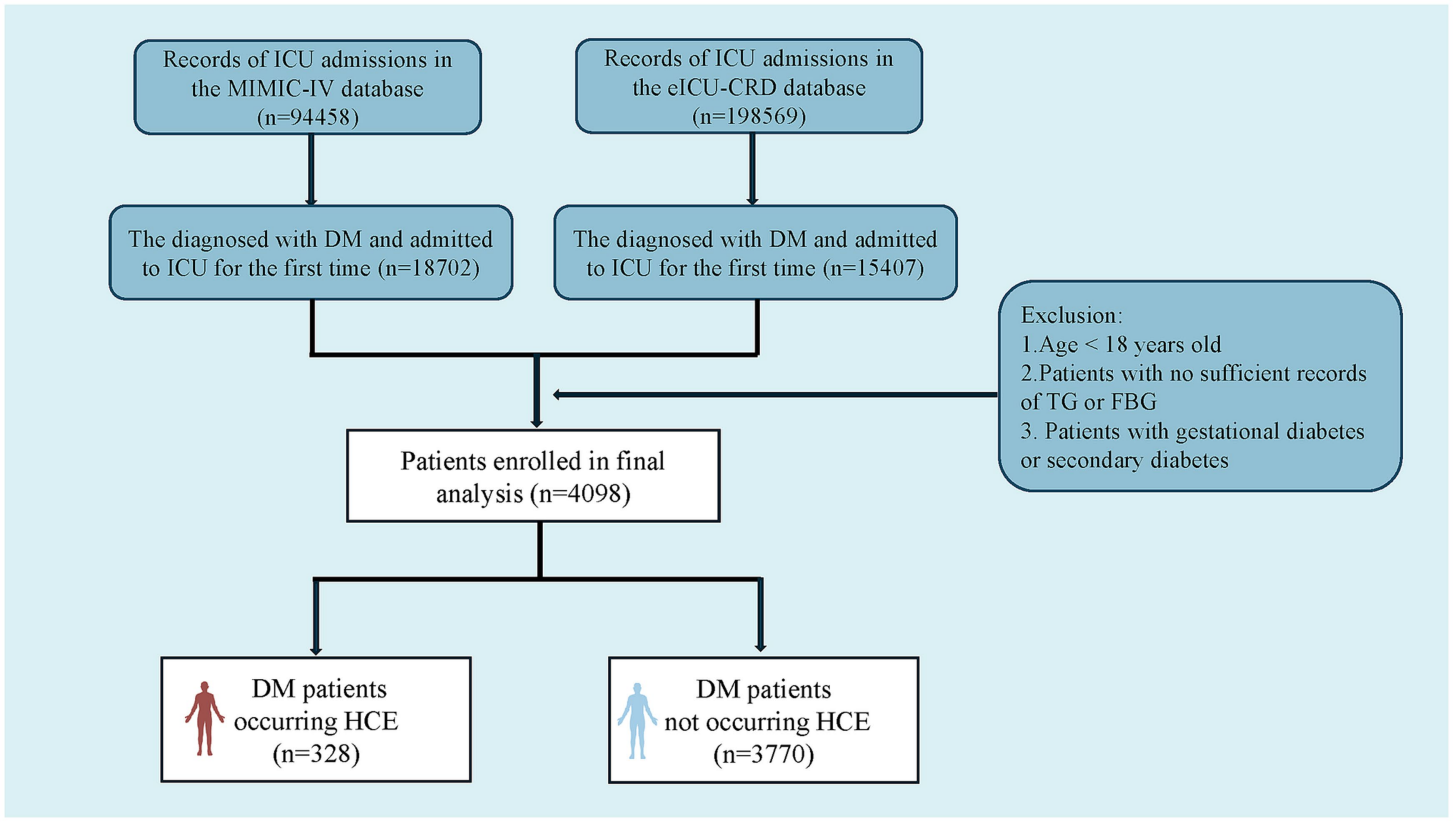
Flowchart of patient selection from the eICU-CRD and the MIMIC-IV databases. TG, triglyceride; FBG, fasting blood glucose; DM, diabetes mellitus; HCE, hyperglycemic crisis episodes; eICU-CRD, eICU Collaborative Research Database; MIMIC-IV, Medical Information Mart for Intensive Care-IV.

Figure 1 depicts a diagrammatic representation of the patient screening procedure, encompassing a total of 4,098 patients [median age: 67.0 (IQR 57.0–77.0); 41.8% female (*n* = 1,712)]. Within these instances, 328 cases experienced HCE. Table 1 encapsulates the fundamental traits of critically ill patients with DM who experienced HCE in contrast to those who did not.

**Table 1:** Baseline characteristics between DM patients with and without HCE.

Variable	Overall (*N* = 4,098)	DM patients without HCE (*N* = 3,770)	DM patients with HCE (*N* = 328)	*p*
TyG index	9.4 (8.9–10.0)	9.4 (8.9–9.9)	10.2 (9.6–11.0)	<0.001
Demographics				
Age (years)	67.0 (57.0–77.0)	68.0 (59.0–78.0)	54.0 (42.0–66.0)	<0.001
Female, *n* (%)	1712.0 (41.8)	1,575.0 (41.8)	137.0 (41.8)	0.998
Vital signs				
HR (bpm)	87.0 (75.0–97.0)	87.0 (74.0–96.0)	93.0 (87.0–109.0)	<0.001
RR (bpm)	20.0 (16.0–22.0)	20.0 (16.0–21.0)	20.0 (17.0–23.0)	0.034
MAP (mmHg)	90.0 (81.0–97.0)	90.0 (82.0–98.0)	90.0 (78.5–93.0)	0.004
Comorbidities				
HTN, *n* (%)	1,876.0 (45.8)	1,787.0 (47.4)	89.0 (27.1)	<0.001
HLP, *n* (%)	1,310.0 (32.0)	1,235.0 (32.8)	75.0 (22.9)	<0.001
COPD, *n* (%)	440.0 (10.7)	424.0 (11.2)	16.0 (4.9)	<0.001
HF, *n* (%)	990.0 (24.2)	942.0 (25.0)	48.0 (14.6)	<0.001
IHD, *n* (%)	764.0 (18.6)	710.0 (18.8)	54.0 (16.5)	0.291
MI, *n* (%)	773.0 (18.9)	739.0 (19.6)	34.0 (10.4)	<0.001
AKI, *n* (%)	1,135.0 (27.7)	974.0 (25.8)	161.0 (49.1)	<0.001
CKD, *n* (%)	825.0 (20.1)	774.0 (20.5)	51.0 (15.5)	0.031
Sepsis, *n* (%)	650.0 (15.9)	579.0 (15.4)	71.0 (21.6)	0.003
Treatment				
Glucocorticoid, *n* (%)	543.0 (13.3)	498.0 (13.2)	45.0 (13.7)	0.794
Hypoglycemic drugs, *n* (%)	1,812.0 (44.2)	1,586.0 (42.1)	226.0 (68.9)	<0.001
Mechanical ventilation, *n* (%)	1,669.0 (40.7)	1,558.0 (41.3)	111.0 (33.8)	0.008
Laboratory measurements				
Hb (g/dL)	12.0 (10.5–13.5)	12.0 (10.5–13.5)	12.0 (10.5–13.5)	0.912
Plt (10⁹/L)	226.0 (178.0–290.0)	226.0 (178.0–290.0)	229.0 (182.5–286.0)	0.542
RBC (m/UL)	4.1 (3.6–4.6)	4.1 (3.6–4.6)	4.1 (3.6–4.6)	0.880
WBC (K/UL)	11.2 (8.4–15.5)	11.2 (8.4–15.3)	11.6 (8.3–17.0)	0.114
Potassium (mEq/L)	4.3 (3.9–4.8)	4.3 (3.9–4.8)	4.3 (3.9–4.9)	0.418
Sodium (mEq/L)	140.0 (137.0–143.0)	140.0 (137.0–143.0)	139.5 (136.0–144.0)	0.356
Creatinine (mg/dL)	1.2 (0.9–1.9)	1.2 (0.9–1.9)	1.4 (0.9–2.3)	<0.001
Urea nitrogen (mg/dL)	24.0 (16.0–43.0)	24.0 (16.0–42.0)	26.0 (16.0–48.5)	0.141
HDL-C (mg/dL)	40.0 (32.0–46.0)	40.0 (32.0–46.0)	40.0 (32.0–44.5)	0.765
TC (mg/dL)	150.0 (119.0–169.0)	150.0 (118.0–168.0)	150.0 (132.0–185.5)	<0.001
Clinical scores				
SOFA	3.0 (1.0–5.0)	3.0 (1.0–5.0)	3.0 (1.0–6.0)	0.486

TyG, triglyceride-glucose; HR, heart rate; RR, respiratory rate; MAP, mean arterial pressure; HTN, hypertension; HLP, hyperlipoidemia; COPD, chronic obstructive pulmonary disease; HF, heart failure; IHD, ischemic heart disease; MI, myocardial infarction; AKI, acute kidney injury; CKD, chronic kidney disease; HB, hemoglobin; PLT, platelet; RBC, red blood cell; WBC, white blood cell; HDL-C, high-density lipoprotein cholesterol; TC, total cholesterol.

Strikingly, critically ill subjects with diabetes manifesting HCE demonstrated considerably amplified TyG index concentrations versus non-HCE counterparts [10.2 (9.6–11.0) vs. 9.4 (8.9–9.9)]. Subsequent logistic regression (Table 2) evaluated TyG’s linkage to HCE propensity among diabetics. The crude model identified TyG as a substantial HCE hazard determinant (OR 2.510; 95% CI: 2.230–2.831). This relationship endured following adjustment for covariates spanning age, sex, HTN, HLP, COPD, HF, MI, AKI, CKD, and sepsis (OR 1.795; 95% CI: 1.576–2.049). Within the fully adjusted Model 3, elevated TyG maintained significant incident HCE linkage (OR 1.806; 95% CI: 1.556–2.101).

**Table 2:** Logistic regression analyses for the correlation between the TyG index and the occurrence of HCE among DM populations.

TyG index	OR	95% CI	*p*
Model 1	2.510	2.230–2.831	<0.001
Model 2	1.795	1.576–2.049	<0.001
Model 3	1.806	1.556–2.101	<0.001

TyG, triglyceride-glucose; HCE, hyperglycemic crisis; DM, diabetes. Model 1: Unadjusted. Model 2: Adjusted for age, gender, hypertension; hyperlipoidemia; chronic obstructive pulmonary disease; heart failure; myocardial infarction; acute kidney injury; chronic kidney disease; sepsis. Model 3: Adjusted for age, gender, hypertension; hyperlipoidemia; chronic obstructive pulmonary disease; heart failure; myocardial infarction; acute kidney injury; chronic kidney disease; sepsis; hypoglycemic drugs; mechanical ventilation; heart rate; respiratory rate; mean arterial pressure; creatinine and total cholesterol.

**Figure 2:**
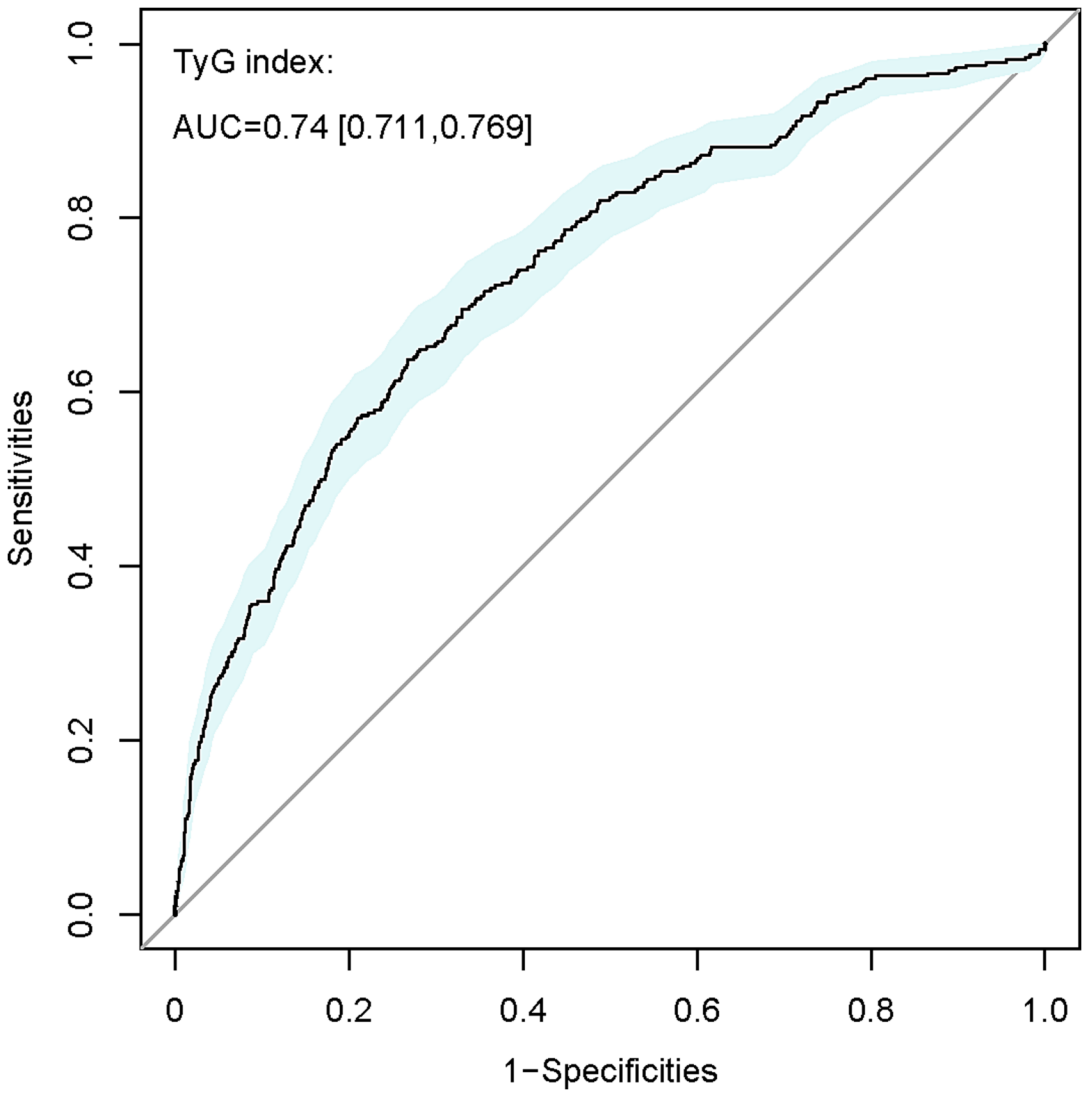
ROC curve of TyG index for identifying patients diagnosed with HCE from the overall DM population. TyG, triglyceride-glucose; AUC, area under the curve.

Additionally, the receiver operating characteristic (ROC) curve analysis (refer to Figure 2) revealed TyG’s effective predictive value for HCE in severely ill patients with DM (AUC 0.740, 95% CI: 0.711–0.769). Its predictive performance surpassed that of age, sex, HR, RR, MAP, HTN, HLP, COPD, HF, IHD, MI, AKI, CKD, sepsis, glucocorticoid therapy, hypoglycemic drug therapy, mechanical ventilation, TC, creatinine, and SOFA score (Supplementary Table S2).

To sum up, the research verifies a positive correlation between increased TyG levels and the risk of HCE in severely ill patients with DM, indicating the TyG index as a possible forecaster of HCE in this group.

### Baseline characteristics and clinical outcomes of the DM-HCE population across different TyG tertiles

We further examined TyG index linkages with clinical sequelae in individuals with DM-HCE. Supplementary Tables S3, S4 document that among 328 critically ill DM subjects developing HCE, 47 succumbed during hospitalization, while 34 expired within ICU confinement. Conspicuously, TyG concentrations proved substantially depressed among hospital discharge survivors [10.1 (9.5–10.8) versus 11.0 (10.2–11.8)] and ICU transfer survivors [10.2 (9.5–10.8) versus 11.5 (10.4–12.0)] relative to corresponding mortality cohorts. Multiple additional baseline parameters diverged considerably between survivor and fatality groups (Supplementary Tables S3, S4). Subsequently, we stratified HCE cases into three cohorts per admission TyG index tertiles (Table 3). Findings indicated that elevated-TyG cohort members demonstrated a propensity for diminished age alongside augmented Hb, RBC, potassium, creatinine, and TC concentrations, coupled with depressed HDL-C levels. Moreover, the uppermost TyG tertile manifested heightened comorbid CKD prevalence.

**Table 3:** Baseline characteristics of DM patients who occurred HCE stratified according to TyG tertiles.

Variable	T1 (*N* = 110)	T2 (*N* = 109)	T3 (*N* = 109)	*p*
TyG index	9.3 (9.0–9.6)	10.2 (10.0–10.4)	11.4 (11.0–11.9)	<0.001
Demographics				
Age (years)	57.0 (44.0–70.0)	56.0 (43.0–65.0)	51.0 (40.0–63.0)	0.036
Female, *n* (%)	47.0 (42.7)	45.0 (41.3)	45.0 (41.3)	0.969
Vital signs				
HR (bpm)	93.5 (87.0–109.0)	92.0 (85.0–108.0)	95.0 (87.0–111.0)	0.480
RR (bpm)	20.0 (16.0–21.0)	20.0 (17.0–24.0)	20.0 (18.0–23.0)	0.091
MAP (mmHg)	90.0 (79.0–92.0)	90.0 (78.0–93.0)	90.0 (79.0–96.0)	0.535
Comorbidities				
HTN, *n* (%)	27.0 (24.5)	31.0 (28.4)	31.0 (28.4)	0.755
HLP, *n* (%)	27.0 (24.5)	29.0 (26.6)	19.0 (17.4)	0.239
COPD, *n* (%)	6.0 (5.5)	7.0 (6.4)	3.0 (2.8)	0.427
HF, *n* (%)	19.0 (17.3)	17.0 (15.6)	12.0 (11.0)	0.398
IHD, *n* (%)	24.0 (21.8)	21.0 (19.3)	9.0 (8.3)	0.016
MI, *n* (%)	14.0 (12.7)	13.0 (11.9)	7.0 (6.4)	0.250
AKI, *n* (%)	50.0 (45.5)	51.0 (46.8)	60.0 (55.0)	0.307
CKD, *n* (%)	20.0 (18.2)	9.0 (8.3)	22.0 (20.2)	0.034
Sepsis, *n* (%)	19.0 (17.3)	22.0 (20.2)	30.0 (27.5)	0.166
Treatment				
Glucocorticoid, *n* (%)	16.0 (14.5)	13.0 (11.9)	16.0 (14.7)	0.801
Hypoglycemic drugs, *n* (%)	74.0 (67.3)	77.0 (70.6)	75.0 (68.8)	0.865
Mechanical ventilation, *n* (%)	35.0 (31.8)	36.0 (33.0)	40.0 (36.7)	0.730
Laboratory measurements				
Hb (g/dL)	11.5 (10.0–12.8)	12.3 (10.8–13.6)	12.4 (10.8–14.0)	0.003
Plt (10⁹/L)	233.0 (195.0–281.0)	229.0 (177.0–291.0)	226.0 (180.0–286.0)	0.882
RBC (m/UL)	3.9 (3.5–4.4)	4.2 (3.7–4.6)	4.2 (3.6–4.7)	0.016
WBC (K/UL)	11.4 (8.1–16.0)	11.9 (8.8–18.2)	11.6 (7.8–16.7)	0.274
Potassium (mEq/L)	4.1 (3.8–4.6)	4.4 (3.9–4.9)	4.4 (4.0–5.0)	0.018
Sodium (mEq/L)	140.0 (136.0–143.0)	139.0 (135.0–144.0)	140.0 (135.0–147.0)	0.332
Creatinine (mg/dL)	1.2 (0.9–1.9)	1.4 (1.0–2.2)	1.7 (1.0–2.7)	0.031
Urea Nitrogen (mg/dL)	25.0 (16.0–43.0)	26.0 (17.0–48.0)	30.0 (15.0–55.0)	0.299
HDL-C (mg/dL)	40.0 (36.0–54.0)	40.0 (33.0–42.0)	39.0 (27.0–40.0)	<0.001
TC (mg/dL)	146.5 (115.0–160.0)	150.0 (140.0–175.0)	150.0 (150.0–234.0)	<0.001
Clinical scores				
SOFA	3.0 (1.0–5.0)	3.0 (1.0–6.0)	3.0 (2.0–7.0)	0.149

**Figure 3:**
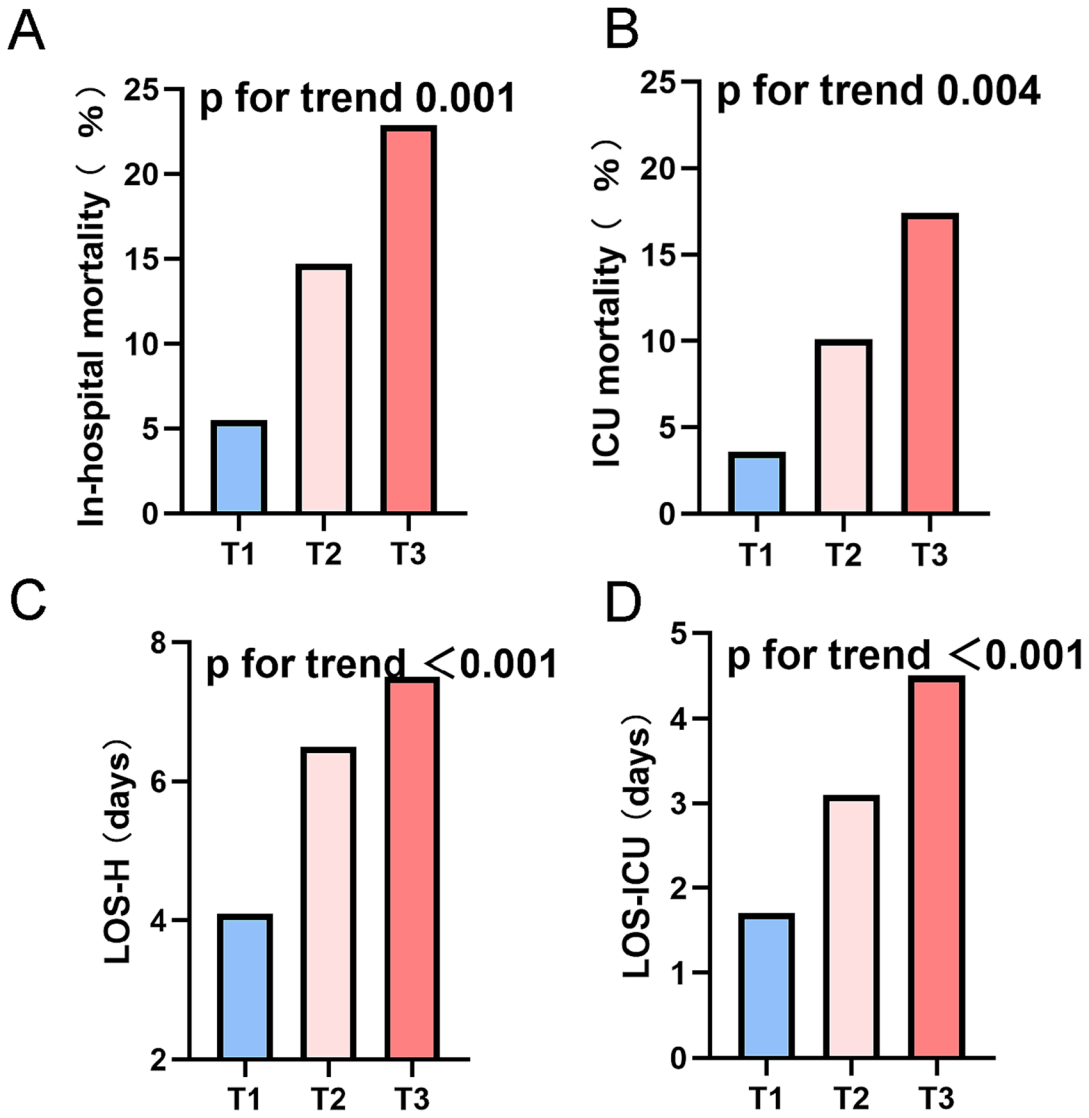
Endpoints stratified by tertiles of the TyG index in the DM-HCE patient population. **(A)** In-hospital mortality. **(B)** ICU mortality. **(C)** LOS-H. **(D)** LOS-ICU. ICU, intensive care unit; LOS-H, length of stay in hospital; LOS-ICU, length of stay in intensive care unit; T, tertile.

Clinical endpoint event comparisons across the three cohorts are itemized in Table 4. Relative to low-TyG subjects, intermediate- and high-TyG groups manifested substantially elevated in-hospital mortality (5.5% vs. 14.7% vs. 22.9%) and ICU mortality (3.6% vs. 10.1% vs. 17.4%), whereas both LOS-H (4.1 vs. 6.5 vs. 7.5 days) and LOS-ICU (1.7 vs. 3.1 vs. 4.5 days) were markedly protracted. Trend scrutiny (Figure 3) confirmed significant mortality ascension—both in-hospital and ICU—with increasing TyG quartile (trend *p* < 0.005). Analogously, LOS-H and LOS-ICU exhibited progressive trajectories across tiers (trend *p* < 0.001).

**Table 4:** Clinical outcome of DM patients who occurred HCE stratified according to TyG tertiles.

Clinical outcomes	T1 (*N* = 110)	T2 (*N* = 109)	T3 (*N* = 109)	*p*
Primary outcomes, *n* (%)				
In-hospital mortality	6 (5.5)	16 (14.7)	25 (22.9)	0.001
ICU mortality	4 (3.6)	11 (10.1)	19 (17.4)	0.004
Secondary outcomes, days				
LOS-H	4.1 (2.9–5.6)	6.5 (4.7–8.0)	7.5 (5.0–13.1)	<0.001
LOS-ICU	1.7 (1.0–2.8)	3.1 (1.7–4.6)	4.5 (3.1–6.4)	<0.001

TyG, triglyceride-glucose; HCE, hyperglycemic crisis; DM, diabetes; LOS-H, length of stay in hospital; LOS-ICU, length of stay in intensive care unit.

Together, these results imply a potential link between a high TyG index and unfavorable outcomes in DM patients suffering from HCE.

### Correlation analysis of TyG index with clinical endpoints in patients with DM-HCE

To probe exhaustively the association binding TyG index and mortality hazard in subjects with DM-HCE, we executed logistic regression analysis (Table 5). Univariate scrutiny disclosed TyG, as a continuous metric, correlated significantly with fatality risk: in-hospital mortality OR = 2.067 (95% CI: 1.555–2.798) and ICU mortality OR = 2.416 (95% CI: 1.744–3.440). Augmented investigation demonstrated that mortality hazard was magnified to 5.159-fold for in-hospital death (95% CI: 2.147–14.412) and 5.594-fold for ICU demise (95% CI: 2.016–19.831) among uppermost versus lowest TyG tertile cohorts. After correcting for confounders, both TyG as a continuous variable (partially corrected model: OR_HOS (hospital)_: 2.038, OR_ICU_: 2.402; fully adjusted model: OR_HOS_: 1.956, OR_ICU_: 2.260) and a categorical variable (T3 vs. T1: model 2: OR_HOS_: 4.367, OR_ICU_: 4.483; model 3: OR_HOS_: 4.374, OR_ICU_: 4.448), this correlation remained statistically significant. The trend analysis revealed a notable gradual increase in mortality risk correlating with rising TyG quartiles (*p*-value below 0.05 across all trends).

**Table 5:** Logistic regression analyses for the correlation between the TyG index and in-hospital mortality in DM populations post-HCE.

Variables	Model 1 OR (95% CI)	*p*	Model 2 OR (95% CI)	*p*	Model 3 OR (95% CI)	*p*
In-hospital mortality						
Per 1 unit increase	2.067 (1.555–2.798)	<0.001	2.038 (1.505–2.819)	< 0.001	1.956 (1.447–2.703)	< 0.001
Tertile 1	Ref	—	Ref	—	Ref	—
Tertile 2	2.982 (1.173–8.600)	0.029	2.871 (1.118–8.346)	0.037	3.155 (1.193–9.472)	0.027
Tertile 3	5.159 (2.147–14.412)	<0.001	4.367 (1.773–12.404)	0.003	4.374 (1.724–12.735)	0.003
*p* for trend	<0.001	—	0.002	—	0.003	—
ICU mortality						
Per 1 unit increase	2.416 (1.744–3.440)	<0.001	2.402 (1.684–3.537)	<0.001	2.260 (1.588–3.331)	<0.001
Tertile 1	Ref	—	Ref	—	Ref	—
Tertile 2	2.975 (0.981–11.013)	0.070	2.842 (0.924–10.635)	0.085	3.106 (0.978–12.026)	0.147
Tertile 3	5.594 (2.016–19.831)	0.003	4.483 (1.563–16.204)	0.010	4.448 (1.503–16.453)	0.012
*p* for trend	0.001		0.008		0.011	

TyG, triglyceride-glucose; HCE, hyperglycemic crisis; DM, diabetes. Model 1: Unadjusted. Model 2: Adjusted for age, gender, acute kidney injury; sepsis. Model 3: Adjusted for age, gender, acute kidney injury; sepsis; mechanical ventilation; SOFA score.

Correlation assessment revealed statistically meaningful associations between TyG concentrations and parameters encompassing LOS-H, LOS-ICU, Hb, RBC, potassium, creatinine, HDL-C, and TC (*p* < 0.05); conversely, no significant statistical linkage emerged with WBC, sodium, or SOFA scores (Supplementary Table S5). Concurrently, multivariate linear regression modeling outcomes established significant correlations relating TyG to LOS-H (*β* = 1.141, *p* < 0.001) and LOS-ICU (*β* = 1.003, *p* = 0.039) (Supplementary Table S6).

The results established a strong link between a high TyG index and unfavorable outcomes, also serving as a standalone risk element for death and extended LOS in patients with DM-HCE.

### Elevated TyG index was associated with higher mortality risk and extended hospitalization duration in patients with DM-HCE

**Figure 4:**
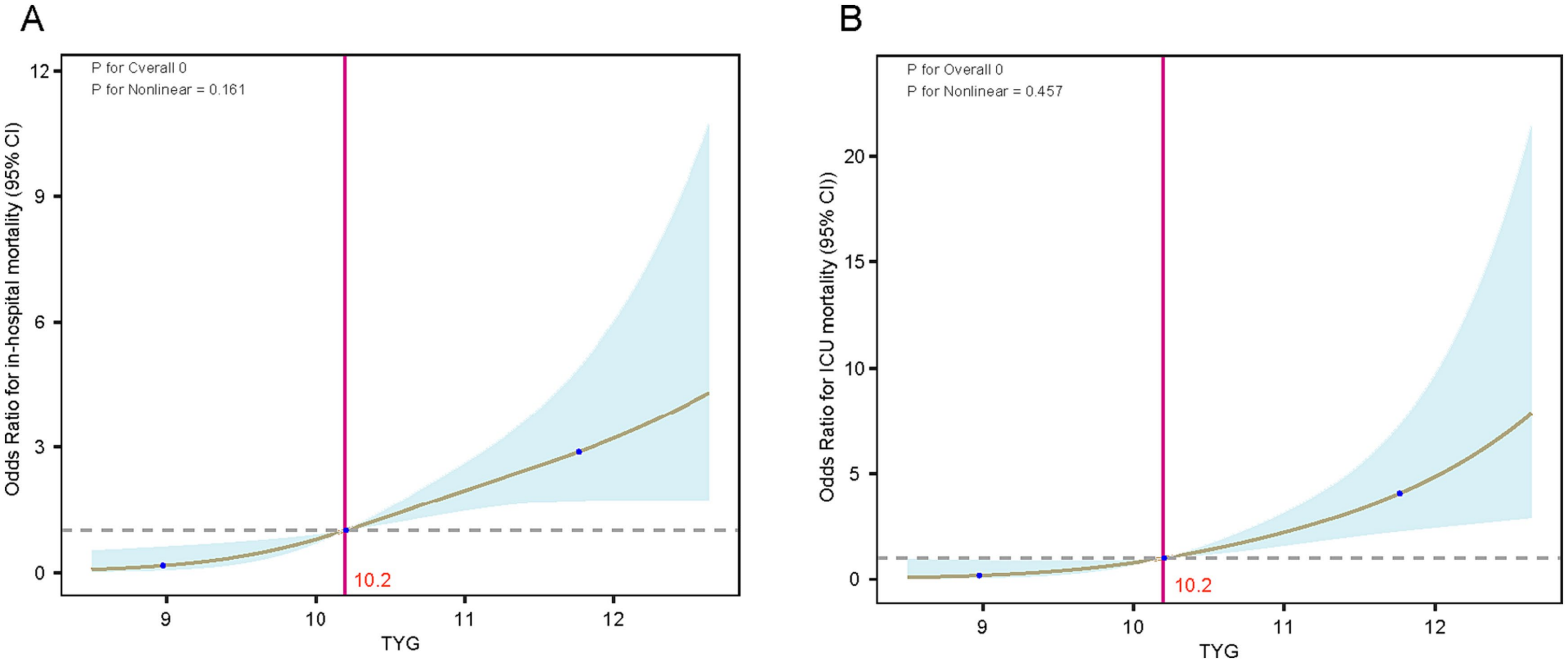
RCS curves of the TyG index in relation to mortality in the DM-HCE patient population. **(A)** In-hospital mortality. **(B)** ICU mortality. ICU, intensive care unit; TyG, triglyceride-glucose; CI, confidence interval.

Post-adjustments for age and gender, the analysis of the RCS curve revealed a notable and direct positive correlation between the TyG index and the likelihood of mortality within the hospital (nonlinear *p* = 0.161) as well as in the ICU (nonlinear *p* = 0.457) for TyG levels exceeding 10.2 (Figure 4). Using the TyG cutoff value of 10.2 identified through RCS analysis, we categorized patients with DM-HCE into distinct groups, with their baseline characteristics comparison presented in Supplementary Table S7. In an effort to mitigate possible confounding factors, we conducted additional analyses using PSM, IPTW, and OW. The initial traits of the group, modified for PSM, IPTW, and OW, are elaborated in Supplementary Tables S8–S10, with most covariates exhibiting a balanced distribution.

**Figure 5:**
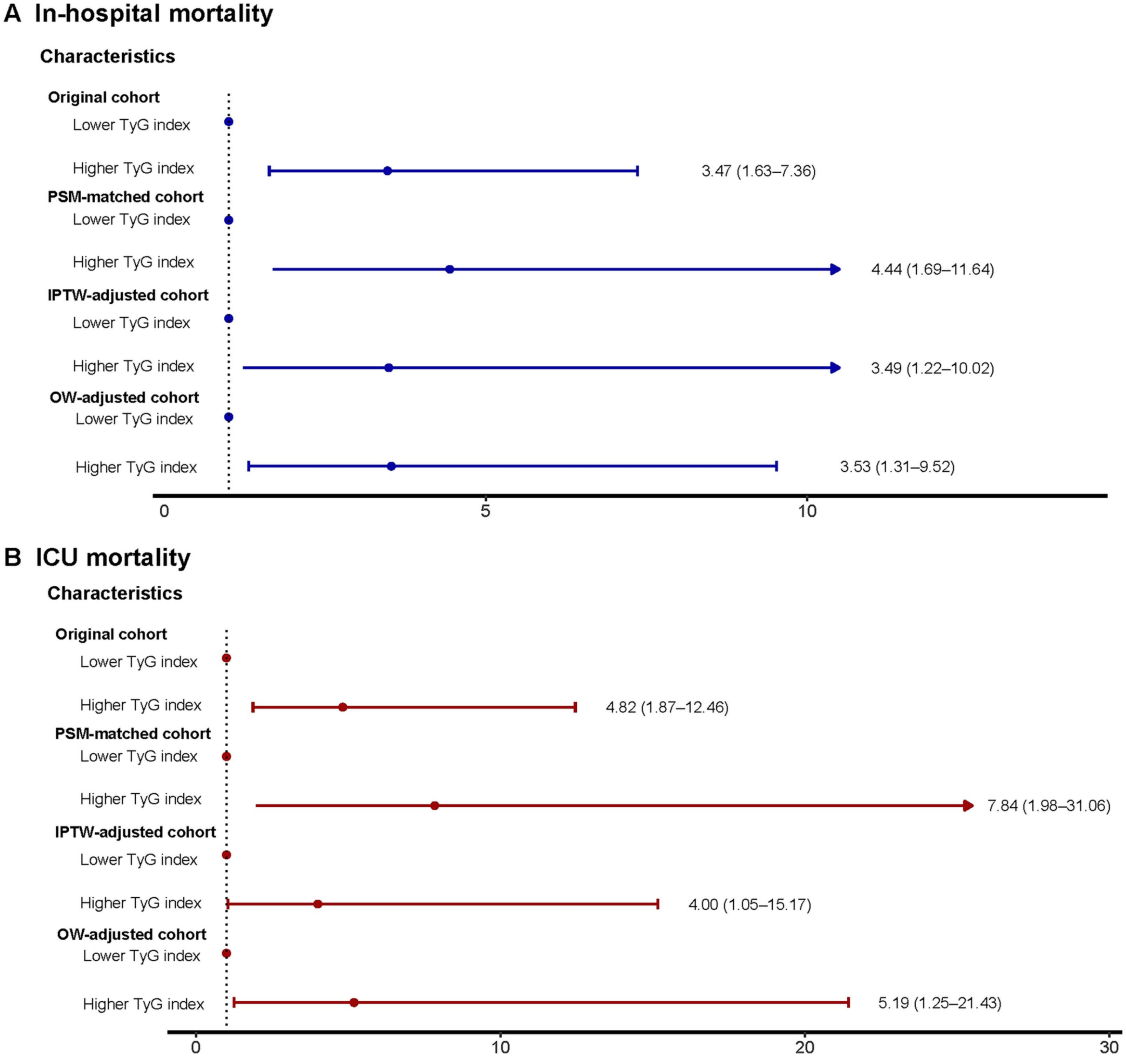
Logistic regression model to explore mortality risks associated with higher TyG index in the original, PSM-, OW-, and IPTW-adjusted cohorts. **(A)** In-hospital mortality. **(B)** ICU mortality. TyG, triglyceride-glucose; ICU, intensive care unit; PSM, propensity score matching; IPTW, inverse probability of treatment weighting; OW, overlap weighting; OR, odds ratio; CI, confidence interval.

Exhibit Supplementary Table S11 reveals that in the initial group, individuals with high TyG levels (>10.2) experienced a notably greater rate of in-hospital occurrences (6.7% vs. 22.0%) and ICU (3.7% vs. 17.1%) mortality rates, and significantly prolonged LOS-H (4.8 days vs. 6.9 days) and LOS-ICU (1.9 days vs. 4.0 days). This trend was maintained in the PSM-corrected cohort (HOS: 7.4% vs. 24.5%, ICU: 2.1% vs. 17.0%), IPTW-corrected cohort (HOS: 6.6% vs. 17.4%, ICU: 4.3% vs. 13.2%), and OW-corrected cohort (HOS: 5.9% vs. 17.1%, ICU: 2.7% vs. 11.6%) all maintained statistical significance. Notably, the results of the analysis of length of hospitalization also showed consistent regularity: the PSM-corrected cohort (LOS-H: 4.9 days vs. 6.8 days, LOS-ICU: 1.9 days vs. 4.0 days), the IPTW-corrected cohort (LOS-H: 4.4 days vs. 7.3 days, LOS-ICU: 1.7 days vs. 4.0 days), and the OW-corrected cohort (LOS-H: 4.8 days vs. 6.9 days, LOS-ICU: 1.9 days vs. 4.0 days) in the high TyG group showed longer hospitalization cycles. Furthermore, logistic regression analysis revealed a significant link between increased TyG levels and mortality in the hospital and ICU, in both the initial group and those adjusted for PSM, OW, or IPTW (all *p*-values were less than 0.05) (Figure 5).

The aggregated study revealed that, post-adjustment through various statistical techniques, a higher TyG index remained closely and positively linked to a rise in death rates and extended hospital stays in patients with DM-HCE.

### Subgroup analysis

**Figure 6:**
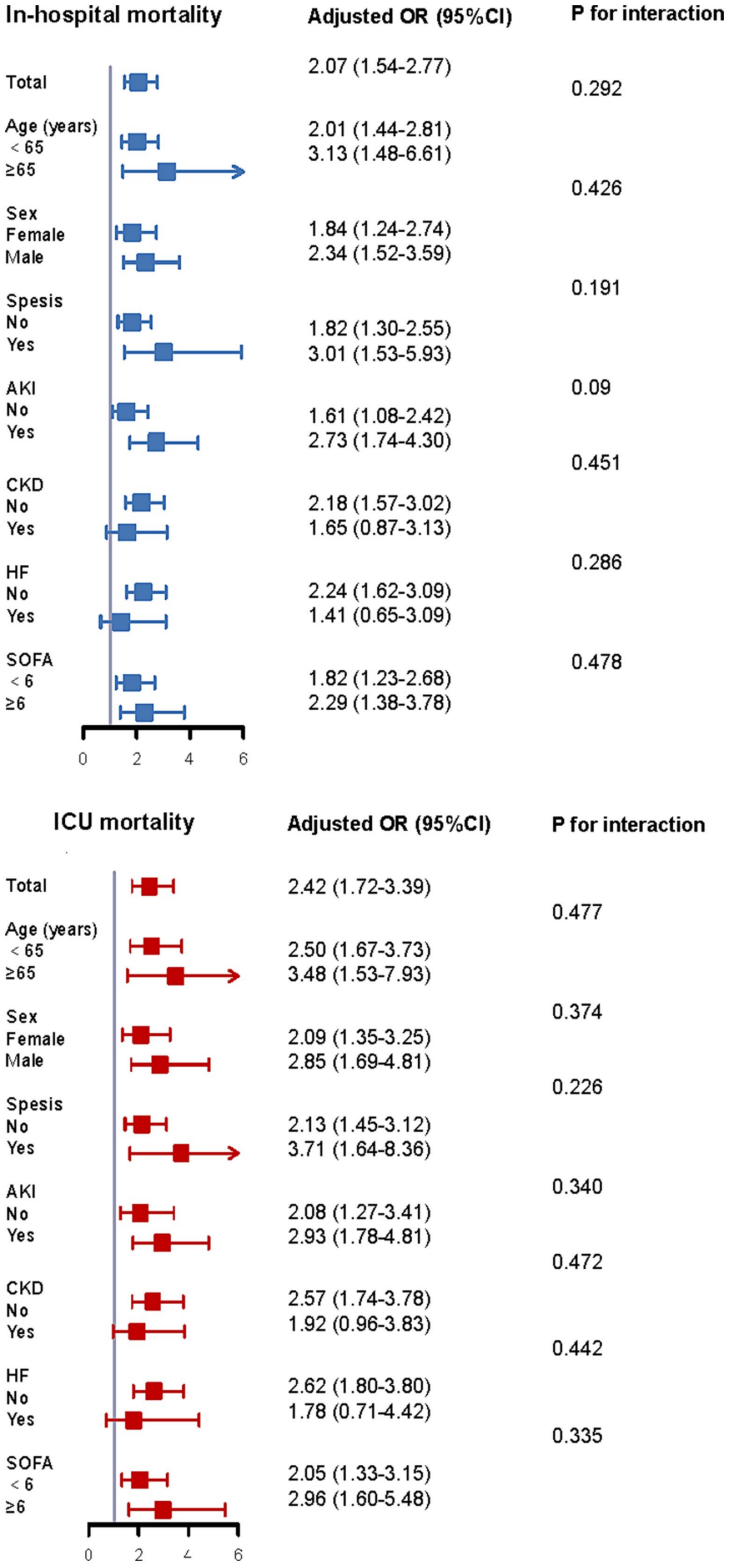
Subgroup analysis of the correlation between TyG index and mortality according to age, sex, comorbidity status, and SOFA score. SOFA, sequential organ failure assessment; ICU, intensive care unit; OR, additive ratio; CI, confidence interval.

Our analysis dichotomized the cohort by age, gender, comorbid conditions, and SOFA metrics to evaluate the TyG index’s prognostic capacity for fatal outcomes across patient strata (Figure 6). Elevated TyG exhibited significant correlation with heightened in-hospital and ICU mortality risk within these cohorts: patients aged <65 years, those ≥65 years, men, women, individuals with or without sepsis, subjects with or without AKI, persons with or without CKD, and non-HF patients. Furthermore, analyses detected no significant interactive effects linking the TyG index to remaining stratification variables (all interaction *p*-values exceeding 0.05).

### Sensitivity analyses

The solidity of our research findings was confirmed by conducting multiple sensitivity analyses. Initially, by omitting 56 patients who had a minimum of one episode of hypoglycemia while in the ICU, the logistic regression analysis outcomes aligned with those of the main analyses (refer to Supplementary Tables S12, S13). Furthermore, by omitting 1,610 participants with incomplete data, the correlation between TyG index and ECM occurrence in severely ill patients with DM, as well as prognosis, aligned with the main results (Supplementary Tables S14, S15). The outcomes of these sensitivity tests validated the dependability and applicability of the primary study’s results.

### Machine learning—TyG and in-hospital mortality

**Figure 7:**
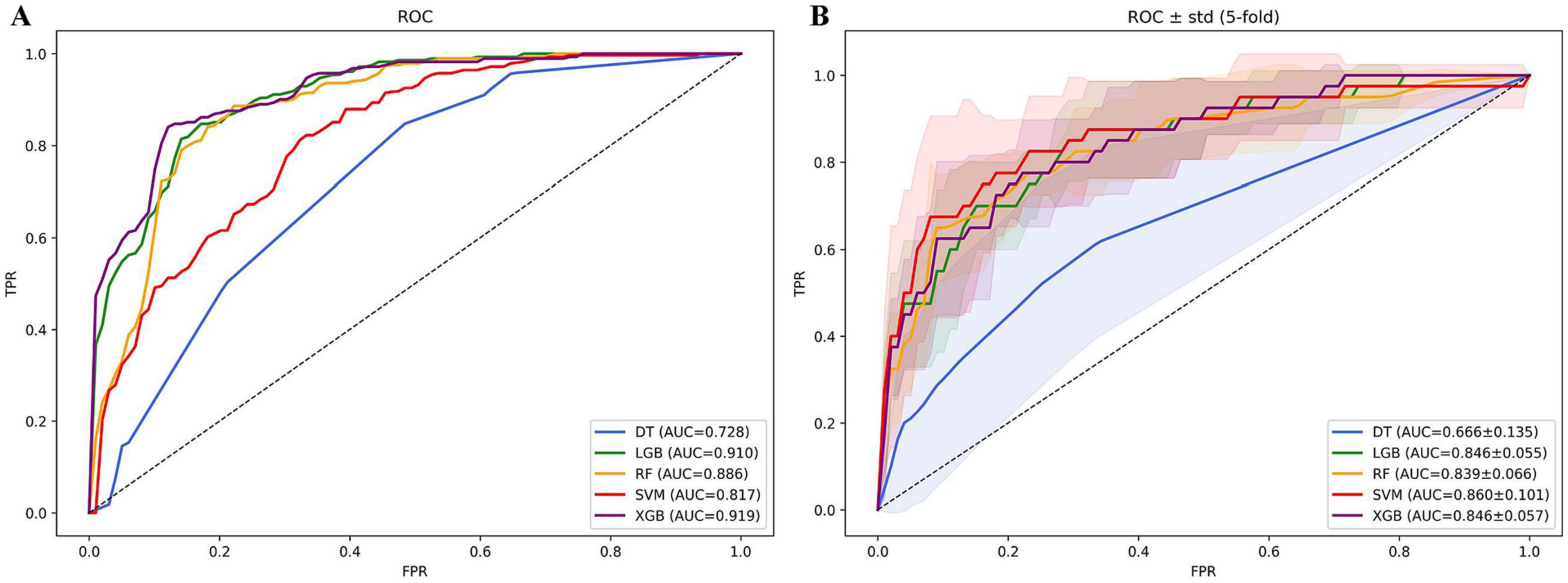
ROC curves of in-hospital mortality prediction models based on machine learning algorithms. **(A)** ROC curve analysis of 5 machine learning algorithms. **(B)** Average AUC performance of 5 machine learning models subjected to 5-fold cross-validation.

Lasso regression selected variables materially contributing to the predictive model’s efficacy. Coefficient fluctuation patterns appear delineated within Supplementary Figure S1. Utilizing these chosen features, we trained and tested five distinct models forecasting in-hospital mortality among subjects with DM-HCE: XGBoost, LGB, RF, SVM, and DT. The XGBoost paradigm exhibited superior predictive capacity (LGB: AUC = 0.910; RF: 0.886; SVM: 0.817; DT: 0.728), securing an AUC of 0.919. This model concurrently manifested elevated sensitivity (0.847), specificity (0.861), accuracy (0.854), and *F*_1_-score (0.853)—detailed in Figure 7A. Counteracting overfitting necessitated five-fold cross-validation; therein, XGBoost sustained peak average performance (AUC = 0.846 ± 0.057), visualized via Figure 7B. Comparative common performance metrics across models populate Supplementary Table S16. DCA curves alongside decision curves for all quintet models reside in Supplementary Figure S2. Consequently, XGBoost emerged as the definitive risk prognostication instrument.

**Figure 8:**
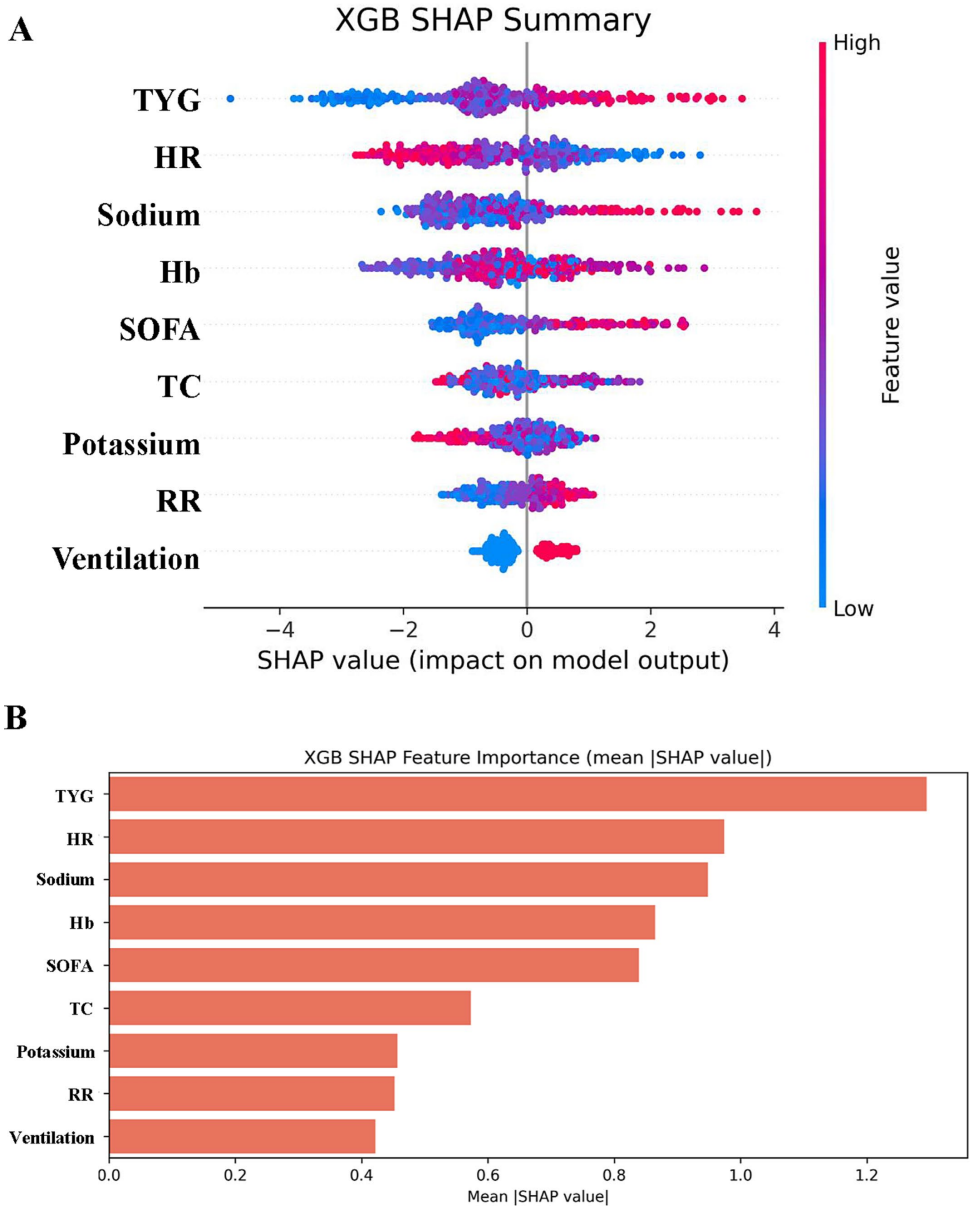
SHAP analysis of XGBoost modeling to predict in-hospital mortality in patients with HCE. **(A)** SHAP summary bar plot. This plot evaluates the contribution of each feature to the model using mean SHAP values, displayed in descending order. **(B)** SHAP summary dot plot. Each dot represents a patient’s SHAP value for a given feature, with red indicating higher feature values and blue indicating lower values. Dots are stacked vertically to show density.

Figure 8 renders a scatter plot of mortality-associated risk factors plus the paramount model’s average importance histogram. The SHAP plot furnishes visual elucidation concerning the predictor variables’ outcome impact. SHAP value magnitude (chromatically encoded) and horizontal axis trajectory (denoting adverse outcome probability) underscore variable effects. Illustratively, both the TyG index and sodium demonstrate expansive chromatic spectrum variance across specimens. These factors concurrently possess the most elongated importance bars situated at the right periphery, mirroring their considerable influence.

## Discussion

This investigation pioneers a methodological appraisal of the TyG index correlations with HCE incidence and clinical trajectories among critically ill diabetic populations, deploying algorithmic risk stratification architectures to refine prognostic precision. Analysis of 4,098 intensive care diabetes cases identified 328 HCE-manifesting individuals exhibiting statistically significant TyG elevations relative to non-HCE counterparts, with TyG demonstrating discriminatory superiority in high-risk cohort identification. Elevated TyG autonomously correlated with escalated fatality rates across hospital and intensive care settings alongside substantially extended LOS durations. These outcomes posit TyG as a premonitory biomarker, furnishing an original paradigm for HCE risk stratification and clinical trajectory governance in critical diabetes care. Machine learning integration substantiates TyG’s centrality within multivariate forecasting frameworks while establishing a clinical decision-support framework for therapeutic interventions.

The TyG index reflects IR status (29, 30), and its association with HCE may be mediated through multiple metabolic and inflammatory pathways (14). IR can induce β-cell dysfunction and excessive free fatty acid release, thereby promoting ketone body production and hyperosmolar states (31). Additionally, elevated TyG levels are often accompanied by enhanced oxidative stress and endothelial dysfunction, which may exacerbate microvascular complications and tissue hypoxia, contributing to HCE development (12, 14, 32–39). Significantly, the TyG index is linked to persistent inflammatory indicators (such as C-reactive protein) and irregularities in neuroendocrine function, both recognized as factors in the advancement of HCE (40, 41). For instance, IR activates the sympathetic nervous system, leading to elevated adrenaline levels that inhibit insulin secretion and worsen hyperglycemia (42). Together, these processes lend biological credibility to the TyG index as an extensive indicator of metabolic imbalance.

Although traditional scoring systems such as SOFA and APACHE-IV are widely used for prognostic assessment in critically ill patients (43, 44), these tools do not directly incorporate glycolipid metabolic parameters. In contrast, the TyG index more precisely captures metabolic dysregulation under acute stress by integrating triglyceride and glucose measurements. Furthermore, its significant association with LOS suggests it may reflect the cumulative systemic organ dysfunction caused by metabolic disturbances. These findings identify a novel target for clinical optimization of HCE management through interventions targeting TyG-related metabolic pathways (e.g., improving insulin resistance), which may reduce mortality and shorten hospital stays.

To minimize confounding bias, this study employed three statistical methods—PSM, IPTW, and OW—for cohort adjustment. Post-adjustment, increased TyG levels continued to show significant correlations with mortality rates in hospitals and ICUs, accompanied by ongoing LOS disparities. The findings are consistent with contemporary research on TyG’s role in predicting sepsis outcomes, affirming its independence from interfering variables. The RCS study additionally confirmed a direct correlation between TyG and the risk of death, suggesting the absence of any limit to its impact on risk and underscoring the necessity for ongoing surveillance in all severely ill sufferers with DM. Subgroup analyses demonstrated stable predictive value across age, gender, and comorbidity status, reinforcing its generalizability.

This study innovatively applied machine-learning algorithms to risk prediction in patients with DM-HCE, with the XGBoost model demonstrating optimal predictive performance (AUC = 0.919). The SHAP analysis indicated that the TyG index was a crucial predictive element of the model, thereby reinforcing its value in clinical decision-making. Compared with traditional statistical models, machine-learning methods exhibit superior capability in capturing nonlinear relationships and interaction effects within high-dimensional data, thereby enhancing predictive accuracy (45–47). These findings establish a foundation for developing intelligent clinical decision support tools in the future. Additionally, the calibration curve of the model showed a strong alignment between forecasted probabilities and real-world observations, and the analysis of the decision curve validated its overall clinical advantage over a broad range, indicating its statistical importance and potential for practical use. The study also identified that beyond the TyG index, SOFA scores and electrolyte levels emerged as key predictive factors, suggesting that HCE patient prognosis may be collectively determined by multiple factors, such as metabolic disorders, organ dysfunction, and electrolyte imbalances.

Nonetheless, this research is subject to multiple constraints. Initially, the backward-looking approach fails to confirm a cause-and-effect link between TyG and HCE, necessitating future validation of the cohort. Second, key details such as insulin use were not recorded, which may have impaired mechanistic elucidation. In addition, the data were limited to 2019, which may not capture the impact of recent therapeutic strategies (e.g., SGLT2 inhibitors) (48). Future studies should explore whether TyG-oriented metabolic management (e.g., insulin intensification or nutritional support) can improve prognosis in intervention trials. Moreover, TyG-based dynamic prediction models (e.g., combined with continuous glucose monitoring) and multicenter randomized controlled trials will be critical to validate its clinical value. Nonetheless, this study provides an innovative framework for metabolic risk monitoring in critically ill patients with diabetes.

## Conclusion

A higher TyG index is closely linked to an increased occurrence and a less favorable outlook for HCE in individuals with DM. Patients with DM-HCE and high TyG levels face a heightened risk of death and extended LOS. For the first time, this research uncovers the medical importance of TyG in forecasting the risk of DM-HCE. Additional comprehensive research is needed to ascertain if early detection and intervention focusing on increased TyG levels can enhance clinical results in this severely ill diabetic group.

## Data Availability

The original contributions presented in the study are included in the article/Supplementary material, further inquiries can be directed to the corresponding authors.
