# Supplementary material for: Correlation of triglyceride-glucose index with the incidence and prognosis of hyperglycemic crises in critically ill patients with diabetes mellitus: a machine-learning-based multicenter retrospective cohort study
Source: Front Nutr. 2025 Sep 4;12:1649553. doi: 10.3389/fnut.2025.1649553 (PMC12443738; doi:10.3389/fnut.2025.1649553)
Supplement: Supplementary file 1 [file Table_1.docx]

**Supplementary materials**

**Table S1. Disease codes included in the study**

| MIMIC-IV | ICD9 | 25000,25001,25002,25012,25013,25040,25041,25042,25043,25050,25051,25052,25053,25060,25061,25062,25063,25070,25072,25080,25081,25082,25090,25092 |
| --- | --- | --- |
|  | ICD10 | E1010,E1021,E1022,E10319,E1040,E1042,E1043,E1051,E10621,E10649,E1065,E109,E1110,E1121,E1122,E11319,E1136,E1139,E1140,E1142,E1143,E1151,E1152,E11610,E11621,E11622, E11628,E11649,E1165,E1169,E118,E119 |
| eICU-CRD | Diagnostic Code ID | 800091,800264,800427,800429,801220,800162,800732,800733,801221,800163,800430 |

**Table S2. AUCs of characteristics for identifying individuals diagnosed with HCE from the overall DM patients**

| **Variables** | **AUC (95% CI)** | ***P*** |
| --- | --- | --- |
| TyG index  Gender  HR  RR  MAP  HTN  HLP  COPD  HF  IHD  MI  AKI  CKD  Sepsis  Glucocorticoid  Hypoglycemic drugs  Mechanical ventilation  TC  Creatinine  SOFA | 0.740 (0.711-0.769)  0.500 (0.472-0.529)  0.665 (0.636-0.694)  0.535 (0.502-0.568)  0.547 (0.515-0.579)  0.601 (0.576-0.627)  0.550 (0.526-0.573)  0.532 (0.519-0.545)  0.552 (0.531-0.572)  0.512 (0.491-0.533)  0.546 (0.529-0.564)  0.616 (0.588-0.644)  0.525 (0.504-0.546)  0.531 (0.508-0.555)  0.503 (0.483-0.522)  0.634 (0.608-0.661)  0.537 (0.511-0.564)  0.580 (0.548-0.611)  0.555 (0.523-0.587)  0.512 (0.479-0.544) | -  < 0.001  < 0.001  < 0.001  < 0.001  < 0.001  < 0.001  < 0.001  < 0.001  < 0.001  < 0.001  < 0.001  < 0.001  < 0.001  < 0.001  < 0.001  < 0.001  < 0.001  < 0.001  < 0.001 |

The *P* value represents the statistical difference in relation to the TyG index.

AUC, area under the curve; CI, confidence interval; HR: heart rate; RR: respiratory rate; MAP: mean arterial pressure; HTN: hypertension; HLP: hyperlipoidemia; COPD: chronic obstructive pulmonary disease; HF: heart failure; IHD: ischemic heart disease; MI: myocardial infarction; AKI: acute kidney injury; CKD: chronic kidney disease; TC: total cholesterol.

**Table 3. Baseline characteristics between survivors and non-survivors among DM populations occurring HCE during hospital stay**

| **Variable** | **Survivors (N = 281)** | **Non-survivors (N = 47)** | ***p*** |
| --- | --- | --- | --- |
| TyG index | 10.1 (9.5 - 10.8) | 11.0 (10.2 - 11.8) | <0.001 |
| Demographics |  |  |  |
| Age (years) | 55.0 (42.0 - 66.0) | 54.0 (44.0 - 66.0) | 0.638 |
| Female, n (%) | 116.0 (41.3) | 21.0 (44.7) | 0.662 |
| Vital signs |  |  |  |
| HR (bpm) | 93.0 (87.0 - 110.0) | 91.0 (80.0 - 106.0) | 0.143 |
| RR(bpm) | 20.0 (17.0 - 22.0) | 21.0 (20.0 - 25.0) | 0.004 |
| MAP (mmHg) | 90.0 (79.0 - 94.0) | 90.0 (73.0 - 91.0) | 0.411 |
| Comorbidities |  |  |  |
| HTN, n (%) | 74.0 (26.3) | 15.0 (31.9) | 0.426 |
| HLP, n (%) | 68.0 (24.2) | 7.0 (14.9) | 0.160 |
| COPD, n (%) | 13.0 (4.6) | 3.0 (6.4) | 0.605 |
| HF, n (%) | 40.0 (14.2) | 8.0 (17.0) | 0.617 |
| IHD, n (%) | 44.0 (15.7) | 10.0 (21.3) | 0.336 |
| MI, n (%) | 30.0 (10.7) | 4.0 (8.5) | 0.652 |
| AKI, n (%) | 131.0 (46.6) | 30.0 (63.8) | 0.029 |
| CKD, n (%) | 42.0 (14.9) | 9.0 (19.1) | 0.462 |
| Sepsis, n (%) | 53.0 (18.9) | 18.0 (38.3) | 0.003 |
| Treatment |  |  |  |
| Glucocorticoid, n (%) | 36.0 (12.8) | 9.0 (19.1) | 0.242 |
| Hypoglycemic drugs, n (%) | 194.0 (69.0) | 32.0 (68.1) | 0.896 |
| Mechanical ventilation, n (%) | 83.0 (29.5) | 28.0 (59.6) | <0.001 |
| Laboratory measurements |  |  |  |
| Hb(g/dL) | 11.9 (10.5 - 13.4) | 12.4 (10.6 - 14.6) | 0.127 |
| Plt(10⁹/L) | 231.0 (184.0 - 284.0) | 223.0 (168.0 - 299.0) | 0.705 |
| RBC(m/Ul) | 4.0 (3.6 - 4.6) | 4.2 (3.7 - 4.7) | 0.274 |
| WBC(K/Ul) | 11.4 (8.0 - 16.1) | 15.0 (9.7 - 21.0) | 0.007 |
| Potassium(mEq/L) | 4.3 (3.9 - 4.9) | 4.4 (3.9 - 4.8) | 0.719 |
| Sodium(mEq/L) | 139.0 (136.0 - 143.0) | 144.0 (137.0 - 152.0) | 0.004 |
| Creatinine(mg/dL) | 1.3 (0.9 - 2.1) | 2.2 (1.3 - 3.9) | <0.001 |
| Urea Nitrogen(mg/dL) | 25.0 (16.0 - 44.0) | 43.0 (16.0 - 66.0) | 0.007 |
| HDL-C(mg/dL) | 40.0 (33.0 - 46.0) | 40.0 (26.0 - 40.0) | 0.042 |
| TC(mg/dL) | 150.0 (132.0 - 186.0) | 150.0 (147.0 - 184.0) | 0.866 |
| Clinical scores |  |  |  |
| SOFA | 3.0 (1.0 - 5.0) | 7.0 (3.0 - 10.0) | <0.001 |

TyG: triglyceride-glucose; HTN: hypertension; HLP: hyperlipoidemia; COPD: chronic obstructive pulmonary disease; HF: heart failure; IHD: ischemic heart disease; MI: myocardial infarction; AKI: acute kidney injury; CKD: chronic kidney disease; Hb: hemoglobin; PLT: platelet; RBC: red blood cell; WBC: white blood cell; HDL-C: high-density lipoprotein cholesterol; TC: total cholesterol.

**Table S4. Baseline characteristics between survivors and non-survivors among DM populations occurring HCE during ICU stay**

| **Variable** | **Survivors (N = 294)** | **Non-survivors (N = 34)** | ***p*** |
| --- | --- | --- | --- |
| TyG index | 10.2 (9.5 - 10.8) | 11.5 (10.4 - 12.0) | <0.001 |
| Demographics |  |  |  |
| Age (years) | 55.0 (42.0 - 66.0) | 52.5 (44.0 - 66.0) | 0.591 |
| Female, n (%) | 120.0 (40.8) | 17.0 (50.0) | 0.304 |
| Vital signs |  |  |  |
| HR (bpm) | 93.0 (87.0 - 110.0) | 95.5 (82.0 - 108.0) | 0.675 |
| RR(bpm) | 20.0 (17.0 - 22.0) | 20.5 (20.0 - 25.0) | 0.022 |
| MAP (mmHg) | 90.0 (79.0 - 94.0) | 90.0 (74.0 - 91.0) | 0.527 |
| Comorbidities |  |  |  |
| HTN, n (%) | 79.0 (26.9) | 10.0 (29.4) | 0.752 |
| HLP, n (%) | 70.0 (23.8) | 5.0 (14.7) | 0.231 |
| COPD, n (%) | 15.0 (5.1) | 1.0 (2.9) | 0.580 |
| HF, n (%) | 42.0 (14.3) | 6.0 (17.6) | 0.600 |
| IHD, n (%) | 46.0 (15.6) | 8.0 (23.5) | 0.241 |
| MI, n (%) | 31.0 (10.5) | 3.0 (8.8) | 0.755 |
| AKI, n (%) | 137.0 (46.6) | 24.0 (70.6) | 0.008 |
| CKD, n (%) | 43.0 (14.6) | 8.0 (23.5) | 0.175 |
| Sepsis, n (%) | 58.0 (19.7) | 13.0 (38.2) | 0.013 |
| Treatment |  |  |  |
| Glucocorticoid, n (%) | 37.0 (12.6) | 8.0 (23.5) | 0.079 |
| Hypoglycemic drugs, n (%) | 205.0 (69.7) | 21.0 (61.8) | 0.342 |
| Mechanical ventilation, n (%) | 89.0 (30.3) | 22.0 (64.7) | <0.001 |
| Laboratory measurements |  |  |  |
| Hb(g/dL) | 12.0 (10.5 - 13.3) | 12.5 (10.6 - 14.7) | 0.064 |
| Plt(10⁹/L) | 227.0 (182.0 - 282.0) | 242.0 (206.0 - 306.0) | 0.331 |
| RBC(m/Ul) | 4.0 (3.6 - 4.5) | 4.2 (3.7 - 4.8) | 0.166 |
| WBC(K/Ul) | 11.4 (7.9 - 16.0) | 17.3 (10.8 - 24.0) | <0.001 |
| Potassium(mEq/L) | 4.3 (3.9 - 4.8) | 4.4 (3.9 - 4.9) | 0.622 |
| Sodium(mEq/L) | 139.0 (135.0 - 143.0) | 145.5 (137.0 - 154.0) | 0.001 |
| Creatinine(mg/dL) | 1.3 (0.9 - 2.2) | 2.3 (1.3 - 3.6) | 0.003 |
| Urea Nitrogen(mg/dL) | 26.0 (16.0 - 46.0) | 47.5 (17.0 - 66.0) | 0.019 |
| HDL-C(mg/dL) | 40.0 (33.0 - 46.0) | 39.0 (25.0 - 40.0) | 0.039 |
| TC(mg/dL) | 150.0 (131.0 - 185.0) | 150.0 (150.0 - 192.0) | 0.198 |
| Clinical scores |  |  |  |
| SOFA | 3.0 (1.0 - 5.0) | 6.5 (3.0 - 10.0) | <0.001 |

**Table S5. Association between the TyG index and several continuous variables**

| **Variable** | **Coef.** | ***p*** |
| --- | --- | --- |
| LOS-H | 0.404 | < 0.001 |
| LOS-ICU | 0.451 | < 0.001 |
| Hb | 0.199 | < 0.001 |
| RBC | 0.150 | 0.007 |
| WBC | 0.0110 | 0.843 |
| Potassium | 0.141 | 0.011 |
| Sodium | 0.080 | 0.147 |
| Creatinine | 0.1224 | 0.0267 |
| HDL-C | -0.293 | < 0.001 |
| TC | 0.330 | < 0.001 |
| SOFA score | 0.087 | 0.117 |

Coef.: coefficient; LOS-H: length of stay in hospital; LOS-ICU: length of stay in intensive care unit.

**Table S6. Multiple linear regression analyses between the TyG index and several continuous variables.**

| **Variable** | **Beta** | **t-value** | ***P*** |
| --- | --- | --- | --- |
| LOS-H | 1.141 | 3.078 | <0.001 |
| LOS-ICU | 1.003 | 1.783 | 0.039 |

Adjusted for age, sex,heart rate, mean arterial pressure, respiratory rate, spesis, hypertension, acute kidney injury, hyperlipidemia, heart failure, myocardial infarction, ischemic heart disease, chronic obstructive pulmonary disease, alanine transaminase, aspartate transaminase, creatinine, lactate, hemoglobin haemoglobin, platelet, red blood cells, white blood cells, potassium, sodium, high density lipoprotein cholesterol, total cholesterol, creatinine, blood urea nitrogen, hypoglycemic drugs, glucocorticoid, mechanical ventilation.

**Table S7. Baseline characteristics between lower and higher TyG index groups in the original population of diabetics with hyperglycemic crisis**

| **Variable** | **Lower TyG index (N = 164)** | **Higher TyG index (N = 164)** | ***p*** |
| --- | --- | --- | --- |
| TyG index | 9.6 (9.2 - 9.9) | 11.0 (10.5 - 11.6) | <0.001 |
| Demographics |  |  |  |
| Age (years) | 57.0 (44.0 - 67.0) | 53.0 (40.0 - 63.5) | 0.019 |
| Female, n (%) | 68.0 (41.5) | 69.0 (42.1) | 0.911 |
| Vital signs |  |  |  |
| HR (bpm) | 91.5 (86.0 - 107.5) | 96.0 (87.0 - 111.0) | 0.107 |
| RR(bpm) | 20.0 (16.0 - 22.0) | 20.0 (17.0 - 23.5) | 0.201 |
| MAP (mmHg) | 90.0 (78.0 - 93.0) | 90.0 (79.0 - 95.5) | 0.427 |
| Comorbidities |  |  |  |
| HTN, n (%) | 42.0 (25.6) | 47.0 (28.7) | 0.535 |
| HLP, n (%) | 42.0 (25.6) | 33.0 (20.1) | 0.237 |
| COPD, n (%) | 8.0 (4.9) | 8.0 (4.9) | >0.999 |
| HF, n (%) | 27.0 (16.5) | 21.0 (12.8) | 0.349 |
| IHD, n (%) | 36.0 (22.0) | 18.0 (11.0) | 0.007 |
| MI, n (%) | 23.0 (14.0) | 11.0 (6.7) | 0.030 |
| AKI, n (%) | 77.0 (47.0) | 84.0 (51.2) | 0.439 |
| CKD, n (%) | 24.0 (14.6) | 27.0 (16.5) | 0.648 |
| Sepsis, n (%) | 31.0 (18.9) | 40.0 (24.4) | 0.228 |
| Treatment |  |  |  |
| Glucocorticoid, n (%) | 20.0 (12.2) | 25.0 (15.2) | 0.422 |
| Hypoglycemic drugs, n (%) | 113.0 (68.9) | 113.0 (68.9) | >0.999 |
| Mechanical ventilation, n (%) | 54.0 (32.9) | 57.0 (34.8) | 0.726 |
| Laboratory measurements |  |  |  |
| Hb(g/dL) | 11.6 (10.2 - 13.1) | 12.4 (10.8 - 13.9) | 0.009 |
| Plt(10⁹/L) | 228.0 (186.5 - 283.5) | 229.5 (182.0 - 290.0) | 0.973 |
| RBC(m/Ul) | 4.0 (3.6 - 4.5) | 4.1 (3.6 - 4.6) | 0.129 |
| WBC(K/Ul) | 11.6 (8.5 - 16.2) | 11.7 (7.9 - 17.3) | 0.866 |
| Potassium(mEq/L) | 4.1 (3.8 - 4.7) | 4.4 (4.0 - 5.0) | <0.001 |
| Sodium(mEq/L) | 139.0 (136.0 - 142.5) | 140.0 (135.5 - 147.0) | 0.112 |
| Creatinine(mg/dL) | 1.2 (0.9 - 1.9) | 1.6 (1.0 - 2.6) | 0.002 |
| Urea Nitrogen(mg/dL) | 25.0 (16.0 - 43.0) | 31.0 (16.5 - 53.5) | 0.035 |
| HDL-C(mg/dL) | 40.0 (35.0 - 52.0) | 40.0 (29.5 - 40.0) | <0.001 |
| TC(mg/dL) | 150.0 (119.0 - 163.5) | 150.0 (150.0 - 218.5) | <0.001 |
| Clinical scores |  |  |  |
| SOFA | 3.0 (1.0 - 5.0) | 3.0 (2.0 - 7.0) | 0.016 |

**Table S8. Baseline characteristics between lower and higher TyG index groups in the PSM-matched DM post-HCE**

| **Variable** | **Lower TyG index (N = 94)** | **Higher TyG index (N = 94)** | ***p*** |
| --- | --- | --- | --- |
| TyG index | 9.6 (9.2 - 9.9) | 10.8 (10.4 - 11.5) | <0.001 |
| Demographics |  |  |  |
| Age (years) | 56.5 (42.0 - 65.0) | 54.0 (41.0 - 66.0) | 0.953 |
| Female, n (%) | 41.0 (43.6) | 39.0 (41.5) | 0.768 |
| Vital signs |  |  |  |
| HR (bpm) | 89.0 (86.0 - 103.0) | 95.5 (86.0 - 110.0) | 0.123 |
| RR(bpm) | 20.0 (16.0 - 21.0) | 20.0 (17.0 - 24.0) | 0.231 |
| MAP (mmHg) | 90.0 (81.0 - 93.0) | 89.0 (74.0 - 90.0) | 0.032 |
| Comorbidities |  |  |  |
| HTN, n (%) | 24.0 (25.5% | 24.0 (25.5) | >0.999 |
| HLP, n (%) | 22.0 (23.4) | 22.0 (23.4) | >0.999 |
| COPD, n (%) | 6.0 (6.4) | 5.0 (5.3) | 0.756 |
| HF, n (%) | 15.0 (16.0) | 12.0 (12.8) | 0.533 |
| IHD, n (%) | 18.0 (19.1) | 14.0 (14.9) | 0.438 |
| MI, n (%) | 10.0 (10.6) | 7.0 (7.4) | 0.446 |
| AKI, n (%) | 50.0 (53.2) | 49.0 (52.1) | 0.884 |
| CKD, n (%) | 15.0 (16.0) | 14.0 (14.9) | 0.840 |
| Sepsis, n (%) | 21.0 (22.3) | 21.0 (22.3) | >0.999 |
| Treatment |  |  |  |
| Glucocorticoid, n (%) | 14.0 (14.9) | 16.0 (17.0) | 0.690 |
| Hypoglycemic drugs, n (%) | 63.0 (67.0) | 65.0 (69.1) | 0.754 |
| Mechanical ventilation, n (%) | 31.0 (33.0) | 32.0 (34.0) | 0.877 |
| Laboratory measurements |  |  |  |
| Hb(g/dL) | 12.0 (10.2 - 13.3) | 12.1 (10.5 - 13.2) | 0.531 |
| Plt(10⁹/L) | 222.5 (176.0 - 272.0) | 221.5 (166.0 - 283.0) | 0.981 |
| RBC(m/Ul) | 4.1 (3.6 - 4.6) | 4.0 (3.5 - 4.4) | 0.530 |
| WBC(K/Ul) | 11.4 (8.1 - 16.3) | 11.2 (7.2 - 17.2) | 0.554 |
| Potassium(mEq/L) | 4.3 (3.9 - 4.9) | 4.4 (4.0 - 4.8) | 0.542 |
| Sodium(mEq/L) | 140.0 (137.0 - 143.0) | 140.0 (135.0 - 147.0) | 0.690 |
| Creatinine(mg/dL) | 1.3 (0.9 - 2.1) | 1.5 (1.0 - 2.4) | 0.220 |
| Urea Nitrogen(mg/dL) | 24.5 (16.0 - 44.0) | 30.5 (17.0 - 52.0) | 0.090 |
| HDL-C(mg/dL) | 40.0 (32.0 - 44.0) | 40.0 (38.0 - 40.0) | 0.556 |
| TC(mg/dL) | 150.0 (128.0 - 174.0) | 150.0 (138.0 - 160.0) | 0.756 |
| Clinical scores |  |  |  |
| SOFA | 3.0 (1.0 - 5.0) | 4.0 (2.0 - 7.0) | 0.060 |

**Table S9. Baseline characteristics between lower and higher TyG index groups in the IPTW-adjusted DM post-HCE**

| **Variable** | **Lower TyG index (N = 341.8)** | **Higher TyG index (N = 328.1)** | ***p*** |
| --- | --- | --- | --- |
| TyG index | 9.7 (9.0 - 10.0) | 10.8 (10.5 - 11.4) | <0.001 |
| Demographics |  |  |  |
| Age (years) | 56.0 (40.9 - 64.2) | 54.3 (41.0 - 67.0) | 0.631 |
| Female, n (%) | 89.9 (36.8) | 133.1 (40.6%) | 0.636 |
| Vital signs |  |  |  |
| HR (bpm) | 95.3 (87.0 - 113.0) | 93.0 (86.0 - 108.0) | 0.524 |
| RR(bpm) | 20.0 (16.0 - 21.0) | 20.0 (17.0 - 24.0) | 0.431 |
| MAP (mmHg) | 87.0 (75.0 - 92.0) | 89.0 (73.0 - 90.0) | 0.782 |
| Comorbidities |  |  |  |
| HTN, n (%) | 97.9 (28.6) | 87.1 (26.6) | 0.804 |
| HLP, n (%) | 103.1 (30.2) | 101.7 (31.0) | 0.928 |
| COPD, n (%) | 15.6 (4.6) | 21.2 (6.5) | 0.585 |
| HF, n (%) | 41.8 (12.2) | 45.2 (13.8) | 0.741 |
| IHD, n (%) | 62.0 (18.1) | 64.3 (19.6) | 0.825 |
| MI, n (%) | 32.3 (9.4) | 31.9 (9.7) | 0.945 |
| AKI, n (%) | 178.2 (52.1) | 184.5 (56.2) | 0.635 |
| CKD, n (%) | 69.8 (20.4) | 67.4 (20.5) | 0.991 |
| Sepsis, n (%) | 107.5 (31.5) | 75.1 (22.9) | 0.341 |
| Treatment |  |  |  |
| Glucocorticoid, n (%) | 62.5 (18.3) | 38.9 (11.9) | 0.344 |
| Hypoglycemic drugs, n (%) | 229.7 (67.2) | 232.8 (71.0) | 0.641 |
| Mechanical ventilation, n (%) | 126.7 (37.1) | 111.4 (33.9) | 0.716 |
| Laboratory measurements |  |  |  |
| Hb(g/dL) | 12.3 (10.4 - 13.5) | 11.9 (9.8 - 13.5) | 0.393 |
| Plt(10⁹/L) | 210.0 (173.0 - 267.0) | 239.0 (183.2 – 280.7) | 0.212 |
| RBC(m/Ul) | 4.1 (3.7 - 4.6) | 4.0 (3.5 - 4.6) | 0.384 |
| WBC(K/Ul) | 10.6 (7.8 - 15.8) | 11.6 (7.2 - 16.9) | 0.624 |
| Potassium(mEq/L) | 4.5 (3.9 - 5.1) | 4.4 (4.0 - 4.9) | 0.742 |
| Sodium(mEq/L) | 140.0 (135.0 - 142.0) | 138.0 (135.0 - 144.0) | 0.873 |
| Creatinine(mg/dL) | 1.3 (0.9 - 2.4) | 1.5 (1.0 - 2.5) | 0.509 |
| Urea Nitrogen(mg/dL) | 26.5 (18.0 - 52.5) | 30.0 (16.6 - 51.6) | 0.896 |
| HDL-C(mg/dL) | 40.0 (35.0 - 44.0) | 40.0 (32.0 - 40.0) | 0.881 |
| TC(mg/dL) | 150.0 (132.0 - 190.7) | 150.0 (133.8 – 170.1) | 0.848 |
| Clinical scores |  |  |  |
| SOFA | 3.0 (1.0 - 7.0) | 3.1 (1.0 - 6.0) | 0.858 |

**Table S10. Baseline characteristics between lower and higher TyG index groups in the OW-adjusted DM post-HCE**

| **Variable** | **Lower TyG index (N = 53.1)** | **Higher TyG index (N = 53.1)** | ***p*** |
| --- | --- | --- | --- |
| TyG index | 9.6 (9.2 - 10.0) | 10.8 (10.5 - 11.3) | <0.001 |
| Demographics |  |  |  |
| Age (years) | 56.0 (42.1 - 65.0) | 54.0 (41.0 - 66.0) | 0.998 |
| Female, n (%) | 22.6 (42.6) | 22.6 (42.6) | >0.999 |
| Vital signs |  |  |  |
| HR (bpm) | 92.0 (86.0 - 109.9) | 95.0 (86.0 - 109.0) | 0.854 |
| RR(bpm) | 20.0 (17.0 - 23.0) | 20.0 (17.0 - 23.0) | 0.747 |
| MAP (mmHg) | 90.0 (76.0 - 93.0) | 90.0 (76.3 - 91.0) | 0.777 |
| Comorbidities |  |  |  |
| HTN, n (%) | 13.0 (24.4) | 13.0 (24.4) | >0.999 |
| HLP, n (%) | 13.8 (26.0) | 13.8 (26.0) | >0.999 |
| COPD, n (%) | 3.0 (5.6) | 3.0 (5.6) | >0.999 |
| HF, n (%) | 7.0 (13.3) | 7.0 (13.3) | >0.999 |
| IHD, n (%) | 9.3 (17.4) | 9.3 (17.4) | >0.999 |
| MI, n (%) | 5.1 (9.5) | 5.1(9.5) | >0.999 |
| AKI, n (%) | 28.0 (52.8) | 28.0 (52.8) | >0.999 |
| CKD, n (%) | 8.8 (16.5) | 8.8 (16.5) | >0.999 |
| Sepsis, n (%) | 11.7 (22.0) | 11.7 (22.0) | >0.999 |
| Treatment |  |  |  |
| Glucocorticoid, n (%) | 7.2 (13.6) | 7.2 (13.6) | >0.999 |
| Hypoglycemic drugs, n (%) | 36.3 (68.4) | 36.3 (68.4) | >0.999 |
| Mechanical ventilation, n (%) | 17.2 (32.3) | 17.2 (32.3) | >0.999 |
| Laboratory measurements |  |  |  |
| Hb(g/dL) | 12.0 (10.2 - 13.4) | 11.9 (10.2 - 13.2) | 0.874 |
| Plt(10⁹/L) | 224.5 (176.5 - 281.8) | 224.0 (179.5 – 280.0) | 0.983 |
| RBC(m/Ul) | 4.1 (3.6 - 4.5) | 4.0 (3.5 - 4.6) | 0.852 |
| WBC(K/Ul) | 11.4 (7.9 - 16.8) | 11.2 (7.1 - 16.8) | 0.581 |
| Potassium(mEq/L) | 4.4 (3.9 – 4.9) | 4.4 (3.9 - 4.8) | 0.797 |
| Sodium(mEq/L) | 140.0 (136.0 - 143.0) | 139.0 (135.0 - 145.0) | 0.748 |
| Creatinine(mg/dL) | 1.3 (0.9 - 2.2) | 1.5 (1.0 - 2.2) | 0.472 |
| Urea Nitrogen(mg/dL) | 27.0 (17.0 - 49.1) | 28.7 (16.7 - 47.8) | 0.801 |
| HDL-C(mg/dL) | 40.0 (33.0 - 44.0) | 40.0 (33.0 - 40.0) | 0.976 |
| TC(mg/dL) | 150.0 (130.1 - 184.3) | 150.0 (138.0 – 166.0) | 0.929 |
| Clinical scores |  |  |  |
| SOFA | 3.0 (1.0 - 6.0) | 3.0 (2.0 - 6.0) | 0.825 |

**Table S11. Clinical outcomes between lower and higher TyG index group in the original, PSM-matched, IPTW-adjusted, and OW-adjusted DM post-HCE populations**

| **Characteristics** | **Lower TyG index** | **Higher TyG index** | ***P*** |
| --- | --- | --- | --- |
| **Original cohort (n = 328)**  In-hospital mortality, n (%)  ICU mortality, n (%)  LOS-H, days  LOS-ICU, days  **PSM-matched cohort (n = 188)**  In-hospital mortality, n (%)  ICU mortality, n (%)  LOS-H, days  LOS-ICU, days  **IPTW-adjusted cohort (n = 669.9)**  In-hospital mortality, n (%)  ICU mortality, n (%)  LOS-H, days  LOS-ICU, days  **OW-adjusted cohort (n = 106.2)**  In-hospital mortality, n (%)  ICU mortality, n (%)  LOS-H, days  LOS-ICU, days | 11.0 (6.7)  6.0 (3.7)  4.8 (3.2 - 6.7)  1.9 (1.0 - 3.1)  7.0 (7.4)  2.0 (2.1)  4.9 (3.4 - 6.9)  1.9 (1.0 - 3.3)  22.7 (6.6)  14.7 (4.3)  4.4 (2.9-6.0)  1.7 (1.2-2.8)  3.1 (5.9)  1.4 (2.7)  4.8 (3.2-6.5)  1.9 (1.0-3.4) | 36.0 (22.0)  28.0 (17.1)  6.9 (4.8 - 11.8)  4.0 (2.3 - 5.7)  23.0 (24.5)  16.0 (17.0)  6.8 (4.7 - 9.5)  4.0 (2.1 - 5.7)  57.1 (17.4)  43.2 (13.2)  7.3 (4.8-12.9)  4.0 (2.6-5.8)  9.1 (17.1)  6.1 (11.6)  6.9 (4.7-12.6)  4.0 (2.2-5.5) | < 0.001  < 0.001  < 0.001  < 0.001  0.001  < 0.001  < 0.001  < 0.001  0.032  0.042  < 0.001  < 0.001  0.010  0.021  < 0.001  < 0.001 |

**Table S12. Logistic regression analyses for the correlation between the TyG index and the occurrence of HCE among DM populations after excluding individuals with hypoglycemic episodes**

| **TyG index** | **OR** | **95％ CI** | ***P*** |
| --- | --- | --- | --- |
| Model 1 | 2.533 | 2.250 – 2.859 | <0.001 |
| Model 2 | 1.847 | 1.619 - 2.111 | <0.001 |
| Model 3 | 2.203 | 1.738 – 2.807 | <0.001 |

TyG: triglyceride-glucose; HCE: hyperglycemic crisis; DM: diabetes.

Model 1: unadjusted

Model 2: Adjusted for age, gender, hypertension; hyperlipoidemia; chronic obstructive pulmonary disease; heart failure; myocardial infarction; acute kidney injury; chronic kidney disease; sepsis.

Model 3: Adjusted for age, gender, hypertension; hyperlipoidemia; chronic obstructive pulmonary disease; heart failure; myocardial infarction; acute kidney injury; chronic kidney disease; sepsis; hypoglycemic drugs; mechanical ventilation; heart rate; respiratory rate; mean arterial pressure; creatinine and total cholesterol.

**Table S13. Logistic regression analyses for the correlation between the TyG index and mortality in DM populations post-HCE after excluding individuals with hypoglycemic episodes**

| **Variables** | **Model 1**  **OR (95％ CI)** | ***P*** | **Model 2**  **OR (95％ CI)** | ***P*** | **Model 3**  **OR (95％ CI)** | ***P*** |
| --- | --- | --- | --- | --- | --- | --- |
| In-hospital mortality | | | | | | |
| Per 1 Unit increase | 2.065 (1.532-2.673) | <0.001 | 2.003 (1.467-2.796) | < 0.001 | 1.879 (1.432-2.611) | < 0.001 |
| Tertile 1 | Ref | - | Ref | - | Ref | - |
| Tertile 2 | 2.846 (1.115-8.290) | 0.027 | 2.795 (1.107-8.052) | 0.031 | 3.008 (1.136-9.040) | 0.027 |
| Tertile 3 | 5.137 (2.027-13.770) | <0.001 | 4.009 (1.681-11.949) | 0.003 | 4.124 (1.624-11.450) | 0.003 |
| *p* for trend | <0.001 | - | 0.001 | - | 0.004 | - |
| ICU mortality | | | | | | |
| Per 1 Unit increase | 2.412 (1.674-3.285) | <0.001 | 2.330 (1.629-3.191) | <0.001 | 2.122 (1.501-3.216) | <0.001 |
| Tertile 1 | Ref | - | Ref | - | Ref | - |
| Tertile 2 | 2.915 (0.834-11.207) | 0.093 | 2.895 (0.929-10.096) | 0.080 | 3.067 (0.976-11.882) | 0.156 |
| Tertile 3 | 5.013 (2.006-18.552) | 0.002 | 4.462 (1.449-16.072) | 0.009 | 4.419 (1.446-16.121) | 0.012 |
| *p* for trend | 0.001 |  | 0.007 |  | 0.013 |  |

TyG: triglyceride-glucose; HCE: hyperglycemic crisis; DM: diabetes.

Model 1: unadjusted

Model 2: Adjusted for age, gender, acute kidney injury; sepsis.

Model 3: Adjusted for age, gender, acute kidney injury; sepsis; mechanical ventilation; sofa score.

**Table S14. Logistic regression analyses for the correlation between the TyG index and the occurrence of HCE among DM populations after** **excluding individuals with any missing value**

| **TyG index** | **OR** | **95％ CI** | ***P*** |
| --- | --- | --- | --- |
| Model 1 | 2.6844 | 2.265 - 3.195 | <0.001 |
| Model 2 | 2.443 | 2.041 - 2.936 | <0.001 |
| Model 3 | 2.261 | 1.811 – 2.837 | <0.001 |

TyG: triglyceride-glucose; HCE: hyperglycemic crisis; DM: diabetes.

Model 1: unadjusted

Model 2: Adjusted for age, gender, hypertension; hyperlipoidemia; chronic obstructive pulmonary disease; heart failure; myocardial infarction; acute kidney injury; chronic kidney disease; sepsis.

Model 3: Adjusted for age, gender, hypertension; hyperlipoidemia; chronic obstructive pulmonary disease; heart failure; myocardial infarction; acute kidney injury; chronic kidney disease; sepsis; hypoglycemic drugs; mechanical ventilation; heart rate; respiratory rate; mean arterial pressure; creatinine and total cholesterol.

**Table S15. Logistic regression analyses for the correlation between the TyG index and mortality in DM populations post-HCE after excluding individuals with any missing value**

| **Variables** | **Model 1**  **OR (95％ CI)** | ***P*** | **Model 2**  **OR (95％ CI)** | ***P*** | **Model 3**  **OR (95％ CI)** | ***P*** |
| --- | --- | --- | --- | --- | --- | --- |
| In-hospital mortality | | | | | | |
| Per 1 Unit increase | 2.335 (1.432-2.656) | 0.002 | 2.105 (1.450-2.893) | < 0.001 | 1.985 (1.405-2.580) | < 0.001 |
| Tertile 1 | Ref | - | Ref | - | Ref | - |
| Tertile 2 | 2.698 (1.130-8.950) | 0.031 | 2.526 (1.134-8.734) | 0.024 | 3.105 (1.124-9.046) | 0.027 |
| Tertile 3 | 5.213 (2.001-14.539) | < 0.001 | 4.969 (1.749-12.151) | 0.003 | 4.443 (1.640-12.171) | 0.002 |
| *p* for trend | < 0.001 | - | 0.002 | - | 0.002 | - |
| ICU mortality | | | | | | |
| Per 1 Unit increase | 2.380 (1.704-3.492) | < 0.001 | 2.498 (1.617-3.449) | < 0.001 | 2.349 (1.574-3.129) | <0.001 |
| Tertile 1 | Ref | - | Ref | - | Ref | - |
| Tertile 2 | 2.559 (0.921-11.400) | 0.081 | 2.838 (0.910-10.178) | 0.093 | 3.152 (0.955-12.337) | 0.151 |
| Tertile 3 | 5.484 (2.002-19.651) | 0.003 | 4.402 (1.525-16.262) | 0.012 | 4.125 (1.474-15.788) | 0.010 |
| *p* for trend | 0.002 |  | 0.009 |  | 0.013 |  |

TyG: triglyceride-glucose; HCE: hyperglycemic crisis; DM: diabetes.

Model 1: unadjusted

Model 2: Adjusted for age, gender, acute kidney injury; sepsis.

Model 3: Adjusted for age, gender, acute kidney injury; sepsis; mechanical ventilation; sofa score.

**Table S16. Comparison of the performance of models in predicting in-hospital mortality rates**

| **Model** | **AUC** | **Sensitivity** | **Specificity** | **Accuracy** | **F1** |
| --- | --- | --- | --- | --- | --- |
| XGBoost | 0.919 | 0.847 | 0.861 | 0.854 | 0.853 |
| LGB | 0.910 | 0.833 | 0.840 | 0.836 | 0.836 |
| SVM | 0.817 | 0.776 | 0.690 | 0.733 | 0.744 |
| DT | 0.728 | 0.847 | 0.516 | 0.681 | 0.727 |
| RF | 0.886 | 0.836 | 0.822 | 0.829 | 0.830 |

XGBoost: Extreme Gradient Boosting; LGB: light gradient boosting machine; DT: decision tree; SVM: support vector machine; RF: random forest


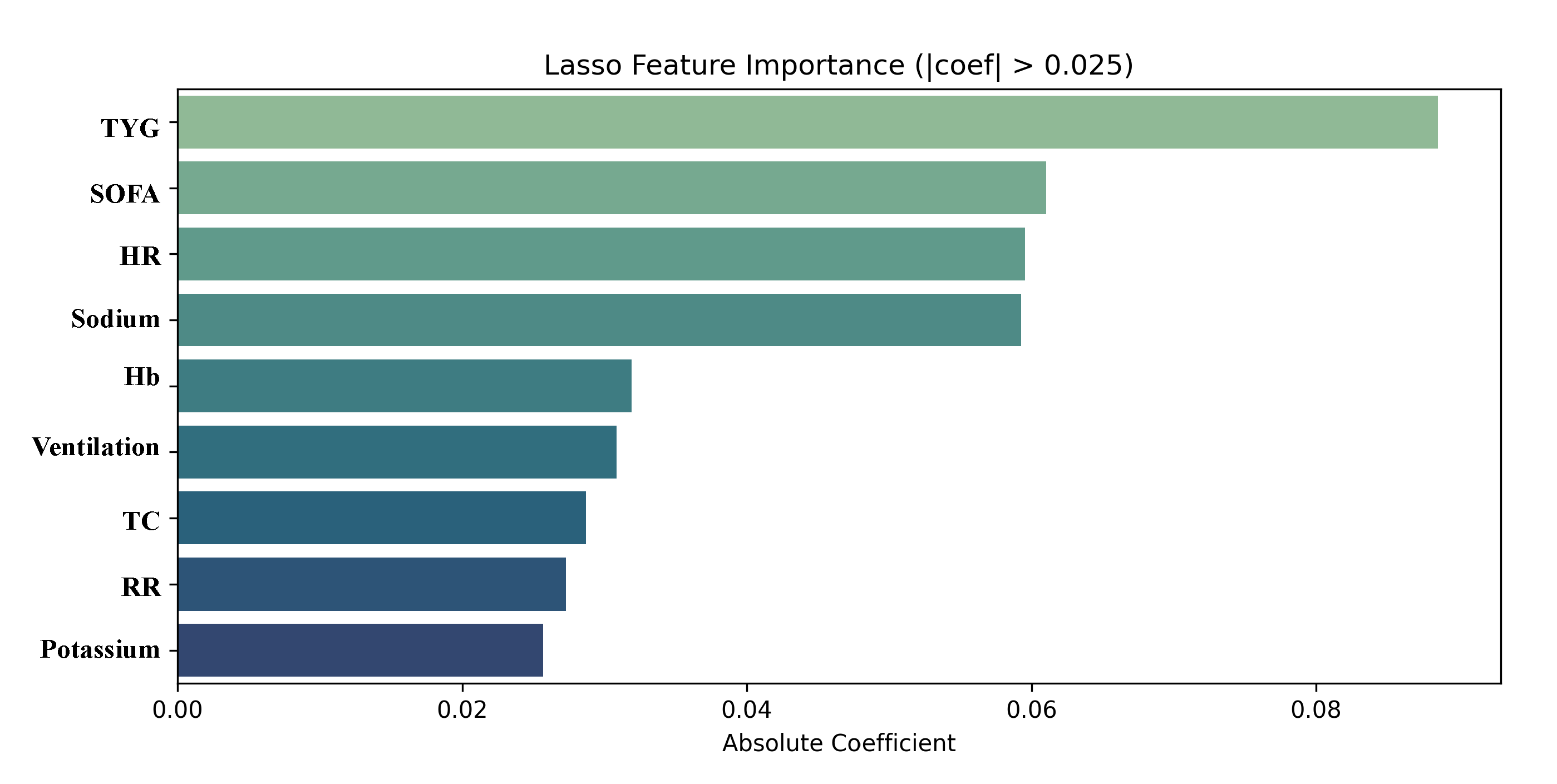
**Fig.S1. Screening of variables based on Lasso regression.**

**Fig.S2.**
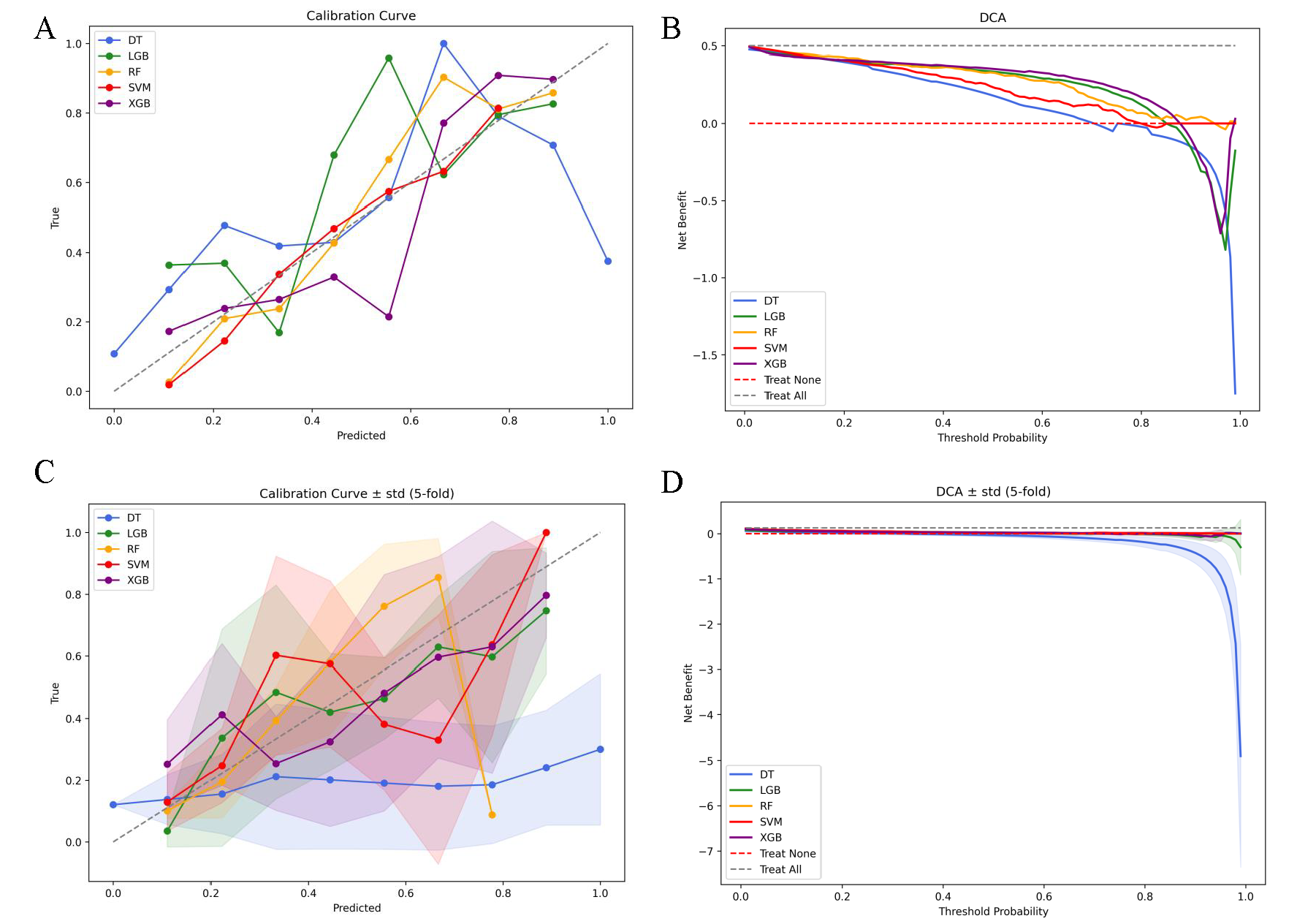
 **Performance of machine learning (ML) models for predicting in-hospital mortality in HCE patients. (A, C) Decision curve analysis curves for each model. (B, D) Calibration curves for multiple models.**
